# Swietemicrolides A–D, mexicanolide-type limonoids from the bark of *Swietenia macrophylla* with *in vitro* cytotoxic and α-glucosidase inhibitory activities†

**DOI:** 10.1039/d4ra01954g

**Published:** 2024-06-11

**Authors:** Tu-Quyen Thi Tran, Duong Hoang Trinh, Binh Thi Dieu Trinh, Dzung Ngoc Bui, Lien-Hoa Dieu Nguyen, Phuong Thu Tran

**Affiliations:** a Faculty of Chemistry, University of Science – Ho Chi Minh City – Vietnam 227 Nguyen Van Cu Street, District 5 Ho Chi Minh City 700000 Vietnam ttphuong@hcmus.edu.vn; b Vietnam National University – Ho Chi Minh City Linh Trung Ward, Thu Duc City Ho Chi Minh City 700000 Vietnam; c Institute of Drug Quality Control 200 Co Bac Street, District 1 Ho Chi Minh City 700000 Vietnam

## Abstract

Four new mexicanolide-type limonoids, swietemicrolides A–D (1–4), together with three known compounds (5–7) were isolated from an ethyl acetate extract of the bark of *Swietenia microphylla*. 1 and 2 had 1,8-hemiacetal systems whilst 3 and 4 shared hexacyclic skeletons consisting of three fused five-membered rings. The structures of the isolated compounds were determined using spectroscopic methods. The five limonoids (1–5) were tested *in vitro* for their cytotoxic effects against two human cancer cell lines (KB carcinoma and A549 lung cancer cells) and α-glucosidase inhibitory activity. None of them showed significant cytotoxic activity, however, swietemicrolide C (3) exhibited strong effect towards α-glucosidase. Moreover, a possible biosynthetic pathway for compounds 1–4 was proposed to support a comprehensive understanding of the configurations of the new limonoids.

## Abstract 2

Four new mexicanolide-type limonoids, swietemicrolides A–D (1–4), together with three known compounds (5–7) were isolated from an ethyl acetate extract of the bark of *Swietenia microphylla*.

## Graphical abstract

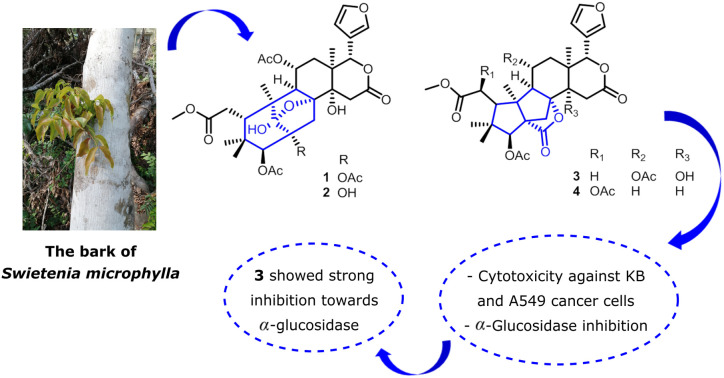

## Introduction

*Swietenia* is a genus belonging to the Meliaceae family, and mainly found in tropical and subtropical Americas. It also grows in China and several Southeast Asian countries such as Vietnam and Indonesia. The genus is well known in the manufacture of furniture, food and traditional medicine and has been reported as a good source of limonoids, steroids, phenolic compounds and sesquiterpenoids.^1^ The compounds have demonstrated a wide range of bioactivities including inhibition of dengue virus 2,^2^ antifeedant,^3–5^ antimicrobial,^6,7^ anti-inflammatory,^8^ hypoglycemic^9–12^ and cytotoxic effects.^13–15^ Limonoids are identified as major natural products which are characteristic of the genus *Swietenia*, especially mexicanolide and phragmalin types.^1,15,16^*Swietenia microphylla* is a new species of the genus *Swietenia*, differing from *Swietenia macrophylla* because of its white grey bark and smaller-sized leaves.^17,18^ It has not been studied for its phytochemical constituents and biological activities although many investigations on the genus have been performed. In Vietnam, the species is planted to cover bare hills and provide wood for the Southeast of Vietnam.

**Fig. 1:**
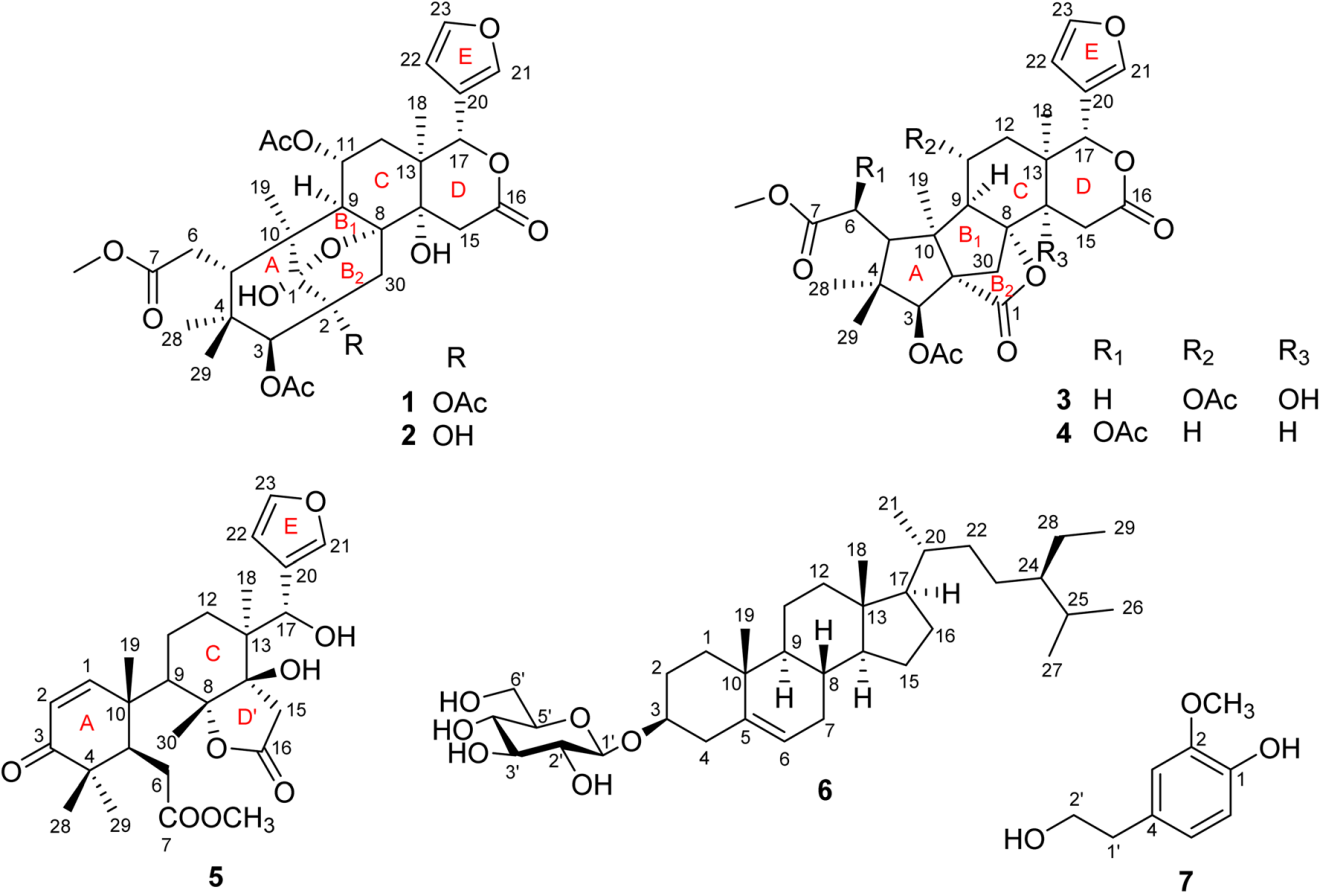
Structure of compounds 1–7.

In the present study, we reported the isolation and structural identification of seven compounds, consisting of four new mexicanolide-type limonoids, named swietemicrolides A–D (1–4), along with a known andirobin-type limonoid characterized as swiemahogin A (5),^19^ a steroid, β-sitosterol-3-*O*-β-d-glucopyranoside (6)^20^ and homovanillyl alcohol (7)^21^ from the bark (Fig. 1). Compounds 3 and 4 were proved to possess a grandifotane scaffold, a complex hexacyclic skeleton containing three fused five-membered rings, which has been seen in a unique compound, grandifotane A, so far.^22^ In addition, the five limonoids (1–5) were evaluated for their *in vitro* biological potency comprising cytotoxic activities against KB carcinoma, A549 lung cancer cell lines and α-glucosidase inhibition.

## Experimental

### General experimental procedures

Melting points were obtained on a Wagner & Munz Polytherm A hot stage microscope. Optical rotations were measured on an A. Krüss Optronic photometer. UV spectra were recorded with an Agilent 8453 spectrophotometer. IR spectra were determined using a Fisher iS50 FT-IR infrared spectrophotometer. NMR spectra were taken on a Bruker Avance III 500 [500 MHz (^1^H) and 125 MHz (^13^C)]. HRESIMS was performed on an X500R QTOF spectrophotometer. Column chromatography (CC) was conducted on silica gel (Merck, 40–63 μm, Merck). Thin layer chromatography (TLC) was developed on precoated TLC plates normal phase 60 F254 (Merck, 250 μm) and RP_18_ (Merck, 200 μm). TLC plates were visualized using UV lamp or spraying with vanillin–H_2_SO_4_ in EtOH and were then heated at about 120 °C for 3–5 min.

### Plant material

The bark of *Swietenia microphylla* was collected in Trang Bom Botanical Garden in January 2019 and identified by Dr Dang Viet Hung, Vietnam National University of Forestry at Dong Nai Province, Vietnam. A voucher sample (SMIB-TB-2019) is deposited in the Natural Product and Medicinal Chemistry Lab, VNUHCM-University of Science.

### Extraction and isolation

The air-dried and ground bark of *S. microphylla* (14 kg) was extracted with EtOAc using a Soxhlet extractor to give an EtOAc extract (E, 890 g). The remaining residue was soaked with MeOH at room temperature (three times, each time in three days), the liquid was then concentrated under vacuum to produce a MeOH extract (M, 2.5 kg). The EtOAc extract was subjected to CC on silica gel with *n*-hexane–acetone (0–100%) as the eluent to afford eight fractions (E1–8). Fraction 5 (103.2 g) was fractionated using CC (silica gel, *n*-hexane–acetone) to yield eight subfractions (E5.1–8). Acetone was added to fraction E5.2 (37.6 g) to produce a precipitate (E5.2B, 454 mg) and a solution (E5.2A, 37.1 g). Fractional CC of E5.2A on silica gel with *n*-hexane–acetone (0–100%) gave five fractions, fraction three was further subjected to CC on silica gel eluted with *n*-hexane–(CHCl_3_–acetone 9 : 1, v/v) yielded nine fractions (A1–9). Purification of fraction A2 using CC (silica gel, *n*-hexane–EtOAc) furnished 4 (6.3 mg). Fraction A4 was separated by CC [silica gel, *n*-hexane–(CHCl_3_–acetone 9 : 1, v/v)] to give eight fractions (A4.1–8). Repeated CC of fractions A4.3, A4.6, A4.7 on silica gel (*n*-hexane–EtOAc) produced 3 (32.3 mg), 1 (75.8 mg) and 2 (11.8 mg). Similarly, fractions A5 and A7 were repeatedly subjected to silica gel CC (*n*-hexane–EtOAc) to obtain 7 (12.9 mg) and 5 (43.4 mg), respectively. Continuous CC of fraction E5.2B (454 mg) on silica gel (*n*-hexane–EtOAc) led to the isolation of 6 (297.5 mg).

#### Swietemicrolide A (1)

Small white needles, m.p. 155–157 °C; [*α*]_D_^25^ – 44.0 (*c* 0.15, MeOH); UV (EtOH) *λ*_max_ (log *ε*): 208 (3.74) nm; IR (KBr) *ν*_max_: 3478, 2951, 2892, 1742, 1436, 1372, 1301, 1222, 1108, 1027 cm^−1^; ^1^H NMR (CDCl_3_, 500 MHz) and ^13^C NMR (CDCl_3_, 125 MHz): see Tables 1 and 2, respectively; HREIMS *m*/*z* 661.2483 [M − H]^−^ (calcd for C_33_H_41_O_14_ 661.2502).

**Table 1:** ^1^H (500 MHz) NMR data for compounds 1–4 in CDCl_3_ (*δ* in ppm, *J* in Hz)

No.	1	2	3	4
1	—	—	—	—
2	—	—	—	—
3	5.52 s	4.93 br s	5.36 s	5.44 s
5	2.73 d (10.1)	2.71 d (10.2)	2.68 dd (10.4, 2.5)	2.73 d (4.8)
6	*H*_a_: 2.66 d (16.7)	*H*_a_: 2.66 d (16.5)	*H*_a_: 2.44 d (10.3)	5.19 d (4.9)
	*H*_b_: 2.35 dd (16.6, 10.1)	*H*_b_: 2.33 dd (16.6, 10.3)	*H*_b_: 2.42 d (2.4)	
9	2.38 d (12.0)	2.46 d (11.9)	2.35 dd (11.1, 2.5)	1.77 dd (5.5, 1.9)
11	5.06 td (11.4, 4.0)	5.05 td (11.4, 3.9)	5.10 td (11.3, 4.5)	*H*_a_: 1.88 ddd (13.3, 5.4, 3.5)
				*H*_b_: 1.64 td (13.5, 2.9)
12	*H*_a_: 2.25 dd (13.7, 4.0)	*H*_a_: 2.25 dd (13.7, 3.7)	*H*_a_: 2.09 dd (13.7, 4.3)	*H*_a_: 1.83 dd (12.5, 2.9)
	*H*_b_: 1.65 dd (13.8, 10.8)	*H*_b_: 1.67 dd (13.4, 11.1)	*H*_b_: 1.92 dd (13.7, 11.6)	*H*_b_: 1.41 dd (12.3, 3.7)
14	—	—	—	2.35 br d (7.2)
15	*H*_a_: 3.05 d (19.0)	3.01 s	*H*_a_: 3.10 d (18.4)	*H*_a_: 2.89 dd (19.3, 7.4)
	*H*_b_: 2.82 d (19.1)		*H*_b_: 2.82 d (18.4)	*H*_b_: 2.82 br d (19.2)
17	5.34 s	5.39 s	5.55 s	5.55 s
18	0.93 s	0.92 s	0.99 s	1.05 s
19	1.19 s	1.23 s	1.10 s	1.24 s
21	7.82 s	7.75 s	7.78 s	7.45 br s
22	6.77 s	6.67 br s	6.71 d (0.8)	6.37 dd (1.8, 0.9)
23	7.44 br s	7.41 br s	7.46 t (1.7)	7.46 t (1.7)
28	1.34 s	1.32 s	1.24 s	1.42 s
29	0.72 s	0.71 s	0.96 s	0.96 s
30	*H*_a_: 2.89 d (14.8)	*H*_a_: 2.80 d (14.5)	*H*_a_: 2.39 d (11.0)	*H*_a_: 2.28 dd (11.3, 2.0)
	*H*_b_: 1.91 d (15.6)	*H*_b_: 1.99 m	*H*_b_: 2.31 dd (11.0, 2.5)	*H*_b_: 2.24 d (11.2)
2-OAc	2.11 s	—	—	—
3-OAc	2.13 s	2.10 s	2.08 s	2.09 s
6-OAc	—	—	—	2.16 s
_7-OMe_	3.67 s	3.66 s	3.69 s	3.79 s
11-OAc	1.93 s	1.91 s	2.02 s	—

#### Swietemicrolide B (2)

Small white needles, m.p. 169–171 °C, [*α*]_D_^25^ – 52.1 (*c* 1.1, MeOH). UV (EtOH) *λ*_max_ (log *ε*) 209 (3.14) nm; IR (KBr) *ν*_max_ 3446, 2921, 2851, 1740, 1636, 1457, 1374, 1228, 1111, 1028 cm^−1^; ^1^H NMR (CDCl_3_, 500 MHz) and ^13^C NMR (CDCl_3_, 125 MHz): see Tables 1 and 2, respectively; HREIMS *m*/*z* 619.2385 [M − H]^−^ (calcd for C_33_H_39_O_14_ 619.2396).

**Table 2:** ^13^C (125 MHz) NMR data for compounds 1–4 in CDCl_3_ (*δ* in ppm)

No.	1	2	3	4
1	108.9, C	109.7, C	171.6, C	173.1, C
2	94.4, C	81.0, C	66.1, C	66.3, C
3	75.7, CH	81.3, CH	76.4, CH	76.4, CH
4	39.9, C	38.7, C	44.6, C	45.7, C
5	39.4, CH	39.6, CH	47.2, CH	51.8, CH
6	32.1, CH_2_	32.3CH_2_	32.3, CH_2_	72.1, CH
7	174.1, C	174.3, C	172.6, C	170.2, C
8	81.4, C	82.6, C	94.0, C	92.0, C
9	58.0, CH	58.5, CH	55.8, CH	55.2, CH
10	47.6, C	44.9, C	51.1, C	50.5, C
11	69.0, CH	69.2, CH	69.0, CH	21.8, CH_2_
12	36.2, CH_2_	36.0, CH_2_	34.0, CH_2_	34.2, CH_2_
13	41.8, C	41.8, C	41.2, C	35.3, C
14	71.3, C	71.3, C	71.3, C	42.4, CH
15	37.0, CH_2_	36.9, CH_2_	37.0, CH_2_	28.2, CH_2_
16	169.0, C	170.1, C	168.0, C	169.3, C
17	77.8, CH	78.0, CH	77.8, CH	77.8, CH
18	15.4, CH_3_	15.7, CH_3_	15.9, CH_3_	22.5, CH_3_
19	22.8, CH_3_	21.8, CH_3_	22.8, CH_3_	22.7, CH_3_
20	120.4, C	120.5, C	120.2, C	121.1, C
21	141.7, CH	141.6, CH	141.5, CH	140.6, CH
22	110.2, CH	110.2, CH	109.8, CH	109.5, CH
23	143.0, CH	142.8, CH	143.3, CH	143.6, CH
28	22.5, CH_3_	22.5, CH_3_	26.7, CH_3_	27.5, CH_3_
29	24.5, CH_3_	24.4, CH_3_	27.3, CH_3_	27.9, CH_3_
30	41.1, CH_2_	41.9, CH_2_	41.7, CH_2_	40.0, CH_2_
2-OAc	175.3, C	—	—	—
	22.3, CH_3_	—	—	—
3-OAc	170.6, C	171.5, C	170.1, C	170.1, C
	21.10, CH_3_	21.12, CH_3_	20.9, CH_3_	20.9, CH_3_
6-OAc	—	—	—	170.3, C
	—	—	—	21.0, CH_3_
7-OCH_3_	52.2, CH_3_	52.1, CH_3_	52.1, CH_3_	52.9, CH_3_
11-OAc	169.8, C	169.8, C	169.8, C	—
	21.08, CH_3_	21.07, CH_3_	21.2, CH_3_	—

#### Swietemicrolide C (3)

Small white needles, m.p. 152–154 °C, [*α*]_D_^25^ – 56.5 (*c* 1.1, MeOH); UV (EtOH) *λ*_max_ (log *ε*): 208 (2.95) nm; IR (KBr) *ν*_max_: 3502, 2960, 2920, 2850, 1793, 1741, 1541, 1457, 1363, 1234, 1045, 1029 cm^−1^; ^1^H NMR (CDCl_3_, 500 MHz) and ^13^C NMR (CDCl_3_, 125 MHz): see Tables 1 and 2, respectively; HREIMS *m*/*z* 647.2318 [M + HCOO]^−^ (calcd for C_32_H_39_O_14_ 647.2345).

#### Swietemicrolide D (4)

Small white needles, m.p. 210–212 °C, [*α*]_D_^25^ – 167.0 (*c* 0.5, MeOH), UV (EtOH) *λ*_max_ (log *ε*): 208 (3.17), 283 (1.89) nm; IR (KBr) *ν*_max_: 3447, 2970, 2940, 2360, 1791, 1743, 1541, 1457, 1374, 1232, 1119, 1044 cm^−1^; ^1^H NMR (CDCl_3_, 500 MHz) and ^13^C NMR (CDCl_3_, 125 MHz): see Tables 1 and 2, respectively; HREIMS *m*/*z* 587.2521 [M + H]^+^ (calcd for C_31_H_39_O_11_ 587.2487).

### *In vitro* cytotoxic assay

The cytotoxic activity of the limonoids were determined using the MTT assay described by Mosmann with ellipticine as the positive control.^23^ The KB (CCL-17™) and A549 (CCL-185™) cell lines were derived from the American type culture collection (ATCC). The cell lines were kept in liquid nitrogen, activated and maintained in Dulbecco’s modified eagle medium (DMEM) containing 7–10% fetal bovine serum (FBS) supplement. The cells were cultured in DMEM at a ratio 1 : 3 in 3–5 days and incubated at 37 °C under 5% CO_2_, 98% humidity for 24 h. The stock solutions were dissolved in DMSO at 2 mg mL^−1^. The experiment was conducted with various concentrations of samples (128, 32, 8 and 2 μg mL^−1^) and duplicated for three times.

### α-Glucosidase inhibitory assay

The α-glucosidase inhibitory assay was performed according to the method described by Wan *et al.* with acarbose as the positive control.^24^ α-Glucosidase, *p*-nitrophenyl α-d-glycopyranoside (pNPG) and acarbose were purchased from Sigma-Aldrich. A mixture consisting of samples (120 μL, 1 unit mL^−1^), α-glucosidase (20 μL, 1 unit mL^−1^) and phosphate buffer (0.1 M, pH 6.8) were pre-incubated for 15 min at 37 °C in a 96-well plate, followed by addition of *p*-nitrophenyl α-d-glucopyranoside (5 mM, 20 μL) solution and incubated for further 15 min at 37 °C. The reaction was then stopped by adding 0.2 M Na_2_CO_3_ (80 μL) and the emission of amount of *p*-nitrophenol was determined at 405 nm. The experiment was duplicated for three times. The inhibition was presented by the IC_50_ values.

## Results and discussion

Compound 1 was obtained as small white needles, m.p. 155−157 °C, [*α*]_D_^25^ – 42.0 (*c* 6.2, MeOH). The HRESIMS spectrum showed a molecular ion peak at *m*/*z* 661.2483 [M − H]^−^ (calcd for C_33_H_41_O_14_ 661.2502), with 13 degrees of unsaturation. The UV spectrum exhibited a maximum at 208 nm whilst the IR spectrum indicated characteristic absorptions at 3478 cm^−1^ (O–H), 1742 cm^−1^ (C=O) and 1222 cm^−1^ (C–O). The ^1^H NMR spectrum of 1 (Table 1) displayed the presence of a β-substituted furan ring [*δ*_H_ 7.82 (1H, s), 7.44 (1H, br s) and 6.77 (1H, s)], a methoxy group (*δ*_H_ 3.67, 3H, s), three acetyl moieties (*δ*_H_ 2.13, 2.11 and 1.93, each 3H, s) and four tertiary methyls (*δ*_H_ 1.34, 1.19, 0.93 and 0.72, each 3H, s). The ^13^C NMR (Table 2) and HSQC spectra revealed resonances for 33 carbons including a furan ring (*δ*_C_ 143.0, 141.7, 120.4 and 110.2); five carbonyls (*δ*_C_ 175.3, 174.1, 170.6, 169.8 and 169.0); one methoxy; seven non-hydrogenated sp^3^ carbons comprising those of three monooxygenated tertiary carbons and one hemiacetal carbon (*δ*_C_ 108.9); five methines including three oxygenated ones; four methylenes; and seven methyls with three in the acetyl groups. Analyses of ^1^H and ^13^C NMR data, especially the β-furanyl ring, suggested that 1 was a tetranortriterpenoid or a mexicanolide-type limonoid. The planar structure of 1 was demonstrated by elucidation of ^1^H–^1^H COSY, HSQC and HMBC spectra. The spectroscopic data of 1 were similar to those of ivorenoid D previously isolated from *Khaya ivorensis*,^25^ except for the existence of the hydroxy at C-14 and the acetoxy instead of the hydroxy group attached to C-11 (*δ*_C_ 69.0). The characteristic chemical shifts of C-1 at *δ*_C_ 108.9 and C-8 at *δ*_C_ 81.4 indicated the presence of a 1,8-hemiacetal motif with an ether bridge between C-8 and C-1. The configuration of the 1,8-hemiacetal and substituents on rings A and B has been described in other related mexicanolides.^25–27^ Additionally, the HMBC correlations from a methine proton (*δ*_H_ 5.06, H-11) and methyl protons (*δ*_H_ 1.93) to a carbonyl carbon (*δ*_C_ 169.8) comprehensively pointed to the acetoxy group bonded to the C-11 position. The presence of an acetoxy group assigned at C-3 was determined by cross-peaks between a methine proton at *δ*_H_ 5.52 (*δ*_C_ 75.7, C-3), methyl protons at *δ*_H_ 2.13 (*δ*_C_ 21.1) and a carbonyl carbon at *δ*_C_ 170.6. Comparison of the chemical shifts of C-1 (*δ*_C_ 108.9), C-2 (*δ*_C_ 94.4) and C-3 (*δ*_C_ 75.7) of compound 1 with those of ivorenoid D indicated that the last acetoxy group was consequently located at the oxygenated tertiary carbon C-2 (*δ*_C_ 94.4). In the NOESY spectrum (Fig. 3), NOE correlations between H_3_-18 and H-22/H_b_-15/H_b_-12, between H_b_-12 and H-9 revealed the same α orientation of the protons. As a result, the designated β-substituted furan ring attached to C-17 was confirmed to be α-oriented. Therefore, proton H-17 possessed the β-orientation. The cross-peaks of H-17/H_a_-30 and H_a_-30/H-11 exhibited that these protons were co-facial. Moreover, NOE correlations observed between H-3 and H_3_-28, H_3_-28 and H_b_-6 indicated that they were α-oriented, and consequently the acetoxy group at C-3 was β-oriented. The compound accordingly had structure 1 which is named swietemicrolide A.

**Fig. 2:**
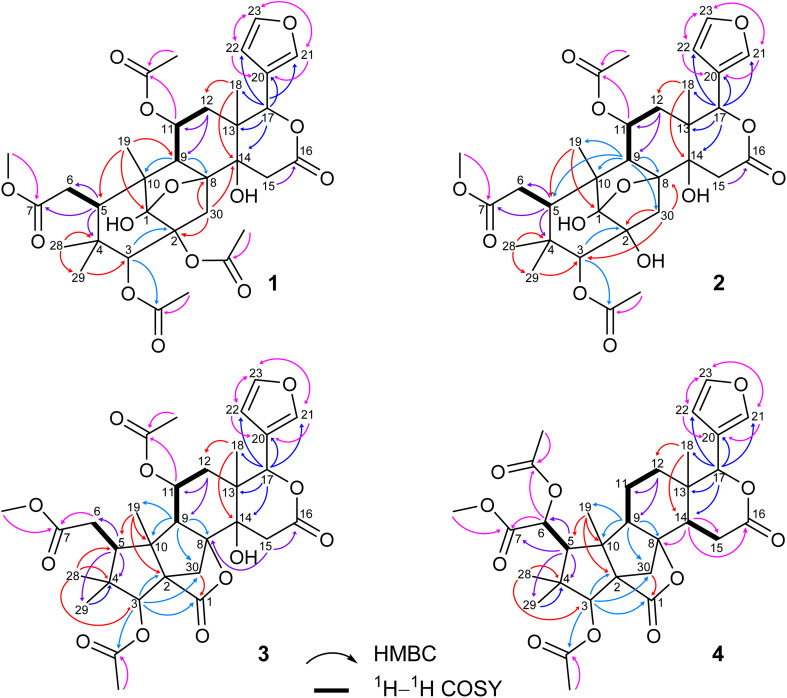
Key HMBC and ^1^H–^1^H COSY correlations of 1–4.

**Fig. 3:**
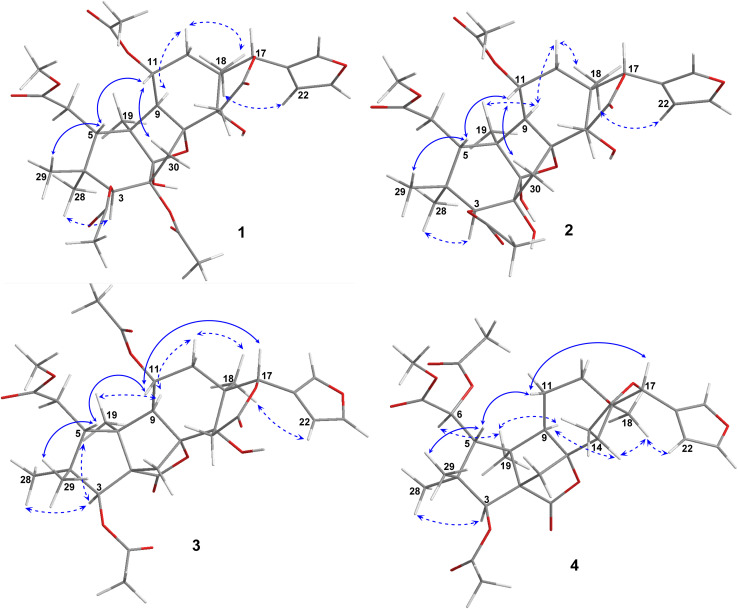
Key NOESY correlations of 1–4.

Compound 2 was isolated as small white needles, m.p. 169–171 °C, [*α*]_D_^25^ – 52.1 (*c* 1.1, MeOH). The UV spectrum showed a maximum at 209 nm and the IR spectrum gave signals at 3446 cm^−1^ (O–H), 1740 cm^−1^ (C=O) and 1228 cm^−1^ (C–O). The 1D (Tables 1 and 2) and 2D (Fig. 2 and 3) NMR spectroscopic data of 2 closely resembled those of 1, suggesting that 2 was also a mexicanolide-type limonoid with a 1,8-hemiacetal system. The significant difference was the absence of an acetoxy group in compound 2. This was in agreement with the HRESIMS spectrum which indicated a deprotonated molecule at *m*/*z* 619.2385, which was assigned the molecular formula of C_31_H_40_O_13_ with one less degree of unsaturation in comparison with that of compound 1. Furthermore, the upfield shift of the oxygenated tertiary carbon (*δ*_C_ 81.0, C-2) in the ^13^C NMR spectrum of 2 implied that the acetoxy group observed in compound 1 was replaced by the hydroxy group. The compound was therefore determined as 2 and is named swietemicrolide B.

Compound 3 was obtained as small white needles, m.p. 152–154 °C, [*α*]_D_^25^ – 56.5 (*c* 1.1, MeOH). It was assigned the molecular formula of C_31_H_38_O_12_ due to its negative peak at *m*/*z* 647.2318 [M + HCOO]^−^ in the HRESIMS spectrum, requiring 13 degrees of unsaturation. The UV spectrum showed a maximum at 208 nm and the IR spectrum had characteristic absorption bands at 3502 cm^−1^ (O–H), 1741 cm^−1^ (C=O) and 1234 cm^−1^ (C–O). The ^1^H and ^13^C NMR spectra of 3 (Tables 1 and 2) revealed resonances for a β-substituted furan ring, a methoxy, two acetyls, four tertiary methyls, four methylenes, five methines, five carbonyl carbons, six non-hydrogenated sp^*3*^ carbons including two oxygenated tertiary carbons. The spectroscopic features suggested that compound 3 possibly was a limonoid. A comparison of the 1D and 2D NMR of compound 3 with those of grandifotane A^22^ indicated that they shared the same skeletal structure. Grandifotane A was isolated from *Khaya grandifoliola*, which was known as the first and the unique mexicanolide-type limonoid bearing grandifotane scaffold. Furthermore, to the best of our knowledge, this is the first time a grandifotane core limonoid has been discovered in the genus *Swietenia*. The main differences were the presence of the acetoxy group bonded to C-11 (*δ*_C_ 69.0) and the methylene (*δ*_H_ 2.44 and 2.42, *δ*_C_ 32.3, C-6) observed instead of the oxymethine. The methylene was determined by the spin coupling system of H_2_-6 and H-5 in ^1^H–^1^H COSY spectrum combined with the key HMBC correlations from H_2_-6 to C-5 and C-7 (Fig. 2). The relative configuration of 3 was partially established by the NOESY spectrum (Fig. 3). The NOE correlations of H_3_-29/H-5, H-5/H-11, H-11/H-17 and H-17/H_a_-12 indicated that they were co-facial and had the β-orientation; whereas those of H_3_-28/H-3, H-3/H_3_-19, H_3_-19/H-9, H-9/H_b_-12, H_b_-12/H_3_-18 and H_3_-18/H-22 assigned these in the α-orientation. The interpretation of NOESY spectrum, NMR data and coupling patterns suggested that relative configuration of 3 has been closely related to that of grandifotane A. The compound was consequently determined to have structure 3 namely swietemicrolide C.

Compound 4 was isolated as small white needles, m.p. 210–212 °C, [*α*]_D_^25^ – 167.0 (*c* 0.5, MeOH), UV: (*λ*_max_) 208 and 283 nm, IR: (*ν*_max_) 3447 cm^−1^ (O–H), 1743 cm^−1^ (C=O) and 1232 cm^−1^ (C–O), C_31_H_38_O_11_ (*m*/*z* 587.2521 [M + H]^+^) with 13 degrees of unsaturation. The 1D (Tables 1 and 2) and 2D (Fig. 2 and 3) NMR spectroscopic data of 4 shared high similarities with those of compound 3 and grandifotane A,^22^ corresponding to the grandifotane skeleton. However, in comparison with grandifotane A, compound 4 contained the methine at C-14 (*δ*_H_ 2.35, *δ*_C_ 42.4) and the acetoxy bonded to C-6. The former was supported by the HMBC correlation between the proton at *δ*_H_ 2.35 and carbons in the *δ*-lactone ring. Meanwhile, the proton H-6 (*δ*_H_ 5.19) correlated to the two carbonyls (*δ*_C_ 170.3 and 170.2). The carbonyl at *δ*_C_ 170.2 showed a cross-peak with methoxy protons (*δ*_H_ 3.79, 3H, s), which was C-7. The remaining carbonyl (*δ*_C_ 170.3) correlated with the methyl protons at *δ*_H_ 2.16, to establish the presence of an acetyl group attached to the oxygenated methine carbon C-6. The correlations observed in the ^1^H–^1^H COSY, HMBC (Fig. 2) and NOESY (Fig. 3) spectra further confirmed the structure and the relative configuration of 4. The structure of compound 4 was therefore characterized to be swietemicrolide D.

**Scheme 1:**
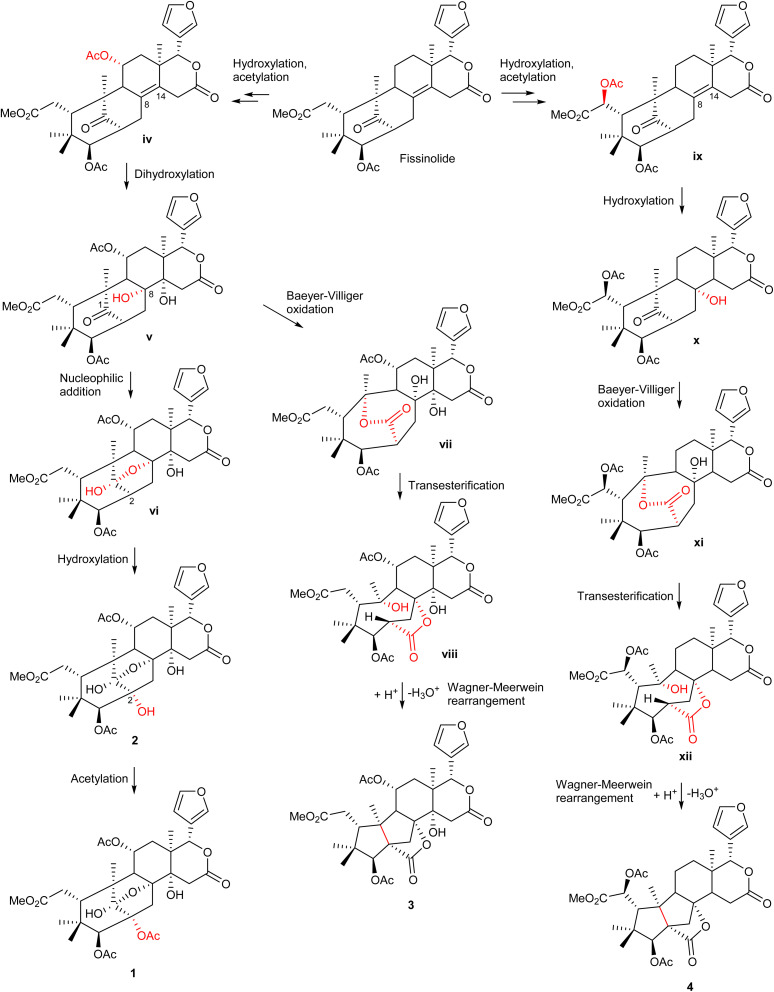
The possible biosynthetic pathways of 1–4.

The possible biosynthetic pathways for 1–4 are proposed in Scheme 1. Fissinolide is likely to be a biogenetic precursor of compounds 1–2, featuring a 1,8-hemiacetal system, and compounds 3–4, which possess a grandifotane scaffold. Hydroxylation at C-11 of fissinolide by a monooxygenase followed by acetylation could result in the formation of iv, which was possibly transformed into v by a dihydroxylation of the Δ^8,14^ double bond to introduce two vicinal hydroxy groups. Compound v could convert into the key intermediate vi*via* an intramolecular nucleophilic addition between the ketonic carbonyl (C-1) and the hydroxy at C-8 to yield the 1,8-hemiacetal linkage. The C-2 of vi could undergo a hydroxylation to form compound 2, which is then likely acetylated to give compound 1. The formation pathways of compounds 3 and 4 is proposed according to the plausible biosynthesis of grandifotane A.^22^ An enzyme-catalyzed Baeyer–Villiger oxidation at the ketonic carbonyl of v would simultaneously result in an ester bridge between C-1 and C-10 in vii, which starts the formation of the unique tricyclodecane ring system. Transesterification followed by protonation of the hydroxy bonded to C-10 and further Wagner–Meerwein rearrangement convert vii*via* the intermediate viii into compound 3 with the removal of a water molecule to form a bond between C-2 and C-10. Similarly, compound 4 could be possibly formed from fissinolide by undergoing a hydroxylation followed by an acetylation at C-6 to give ix. Hydroxylation at the Δ^8,14^ double bond could introduce a hydroxy at C-8 (x). The grandifotane skeleton is likely designated as in 3 through several steps *via* the intermediates xi and xii. The biosynthetic pathway proposal could not only explain the formation of the fused ring systems but also support the relative configuration of 1–4 which are consistent with those of previously reported mexicanolides.^22,28^

The five limonoids, including swietemicrolides A–D (1–4) and swiemahogin A (5), were evaluated for their *in vitro* cytotoxicity against two human cancer cell lines, KB epidermal carcinoma and A549 human lung cancer cells using 3-(4,5-dimethylthiazolyl-2)-2,5-diphenyltetrazolium bromide (MTT) assay described by Mosmann^23^ with ellipticine as the positive control (Table 3). Only swietemicrolide C (3) inhibited more than 50% cell growth at the concentration of 128 μg mL^−1^ and therefore was further tested for the IC_50_ values and displayed weak activities (IC_50_ = 174.8 μM and 189.9 μM, respectively). Moreover, they were accessed for their *in vitro* α-glucosidase inhibitory effects (Table 3).^24^ As the result, only swietemicrolide C (3) showed potential activity with an IC_50_ value of 253.6 μM, about three times stronger than the positive control, acarbose (IC_50_ = 697.7 μM), while the others were found to be completely inactive. The results suggested that the appearance of an acetoxy side chain at C-11 and a hydroxy group at C-14 attached to the grandifotane skeleton increased the effect whilst the presence of an acetoxy group at C-6 could reduce the activity.

**Table 3:** Cytotoxicity and α-glucosidase inhibitory activities of compounds 1–5

Compound	IC_50_ (μM)		
	Cytotoxicity		α-Glucosidase inhibition
	KB (μM)	A549 (μM)	
1	>190	>190	—
2	>200	>200	—
3	174.8 ± 5.9	189.9 ± 4.6	253.6 ± 6.9
4	>128	>128	—
5	>128	>128	—
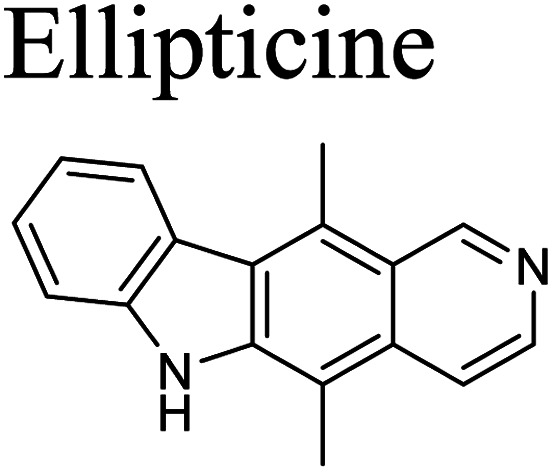	1.79 ± 0.1	1.74 ± 0.1	—
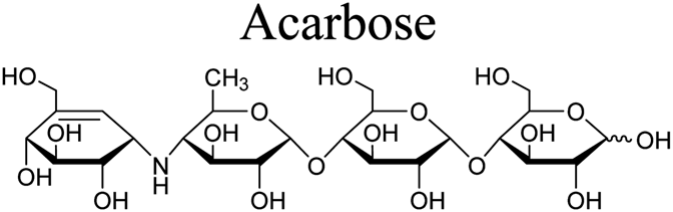	—	—	697.7 ± 62.0

## Conclusions

In summary, four new members of the B,D-ring *seco* limonoids named swietemicrolides A–D (1–4) were isolated from the bark of *S. microphylla*. Compound 3, bearing uncommon three fused five-membered rings, exhibited strong α-glucosidase inhibitory activity and fairly weak cytotoxicity against KB and A549 human cell lines. The study significantly contributed to enriching the structural diversity of limonoid derivatives, especially grandifotane class, and provided target subtances for further investigation of biological activities.

## Data availability

The data supporting this article have been included as part of the ESI.†

## Author contributions

Tu-Quyen Thi Tran: data curation, formal analysis, investigation, methodology, visualization, writing – original draft; Duong Hoang Trinh: formal analysis, investigation, methodology, writing – review and editing; Binh Thi Dieu Trinh: formal analysis, investigation; Dzung Ngoc Bui: formal analysis, investigation; Lien-Hoa Dieu Nguyen: conceptualization, methodology, supervision; Phuong Thu Tran: conceptualization, methodology, validation, supervision, project administration, writing – review and editing.

## Conflicts of interest

There are no conflicts to declare.

## Supplementary Material

RA-014-D4RA01954G-s001

## References

[cit1] Sun Y. P., Jin W. F., Wang Y. Y., Wang G., Morris-Natschke S. L., Liu J. S., Wang G. K., Lee K. H. (2018). Molecules.

[cit2] Cheng Y. B., Chien Y. T., Lee J. C., Tseng C. K., Wang H. C., Lo I. W., Wu Y. H., Wang S. Y., Wu Y. C., Chang F. R. (2014). J. Nat. Prod..

[cit3] Abdelgaleil S. A., Doe M., Morimoto Y., Nakatani M. (2006). Phytochemistry.

[cit4] Fowles R., Mootoo B., Ramsewak R., Khan A., Ramsubhag A., Reynolds W., Nair M. (2010). Pest Manag. Sci..

[cit5] Mootoo B. S., Ali A., Motilal R., Pingal R., Ramlal A., Khan A., Reynolds W. F., McLean S. (1999). J. Nat. Prod..

[cit6] Lin B. D., Yuan T., Zhang C. R., Dong L., Zhang B., Wu Y., Yue J. M. (2009). J. Nat. Prod..

[cit7] Rahman A. K., Chowdhury A. K., Ali H. A., Raihan S. Z., Ali M. S., Nahar L., Sarker S. D. (2009). J. Nat. Med..

[cit8] Chen J. J., Huang S. S., Liao C. H., Wei D. C., Sung P. J., Wang T. C., Cheng M. J. (2010). Food Chem..

[cit9] Dewanjee S., Maiti A., Das A. K., Mandal S. C., Dey S. P. (2009). Fitoterapia.

[cit10] Lau W. K., Goh B. H., Kadir H. A., Shu-Chien A. C., Tengku Muhammad T. S. (2015). Molecules.

[cit11] Maiti A., Dewanjee S., Sahu R. (2009). Phytother. Res..

[cit12] Ovalle-Magallanes B., Medina-Campos O. N., Pedraza-Chaverri J., Mata R. (2015). Phytochemistry.

[cit13] Pudhom K., Sommit D., Nuclear P., Ngamrojanavanich N., Petsom A. (2009). J. Nat. Prod..

[cit14] Sun Y. P., Zhu L. L., Liu J. S., Yu Y., Zhou Z. Y., Wang G., Wang G. K. (2018). Fitoterapia.

[cit15] Sun Y. P., Xie Z., Jin W. F., Liu Y. W., Sun L. J., Liu J. S., Wang G. K. (2024). Org. Biomol. Chem..

[cit16] Lin B. D., Zhang C. R., Yang S. P., Zhang S., Wu Y., Yue J. M. (2009). J. Nat. Prod..

[cit17] https://vafs.gov.vn/vn/ky-thuat-trong-cay-xa-cu-la-nho/, accessed February 2024

[cit18] Sangeetha K., Yasotha P. (2014). Res. Rev.: J. Toxicol..

[cit19] Chen Y. Y., Wang X. N., Q Fan C., Y Yin S., Yue J. M. (2007). Tetrahedron Lett..

[cit20] Mizushina Y., Nakanishi R., Kuriyama I., Kamiya K., Satake T., Shimazaki N., Koiwai O., Uchiyama Y., Yonezawa Y., Takemura M., Sakaguchi K., Yoshida H. (2006). J. Steroid Biochem. Mol. Biol..

[cit21] DellaGreca M., Monaco P., Pinto G., Pollio A., Previtera L., Temussi F. (2001). Bull. Environ. Contam. Toxicol..

[cit22] Yuan T., Zhu R. X., Zhang H., Odeku O. A., Yang S. P., Liao S. G., Yue J. M. (2010). Org. Lett..

[cit23] Mosmann T. (1983). J. Immunol. Methods.

[cit24] Wan L. S., Chen C. P., Xiao Z. Q., Wang Y. L., Min Q. X., Yue Y. D., Chen J. C. (2013). J. Ethnopharmacol..

[cit25] Wu W. B., Zhang H., Liu H. C., Dong S. H., Wu Y., Ding J., Yue J. M. (2014). Tetrahedron.

[cit26] Joseph D. C., Malcolm M., Domingo A. O., David A. T. (1976). J. Chem. Soc., Perkin Trans. 1.

[cit27] Nakatani M., Abdelgaleil S. A., Okamura H., Iwagawa T., Doe M. (2000). Chem. Lett..

[cit28] Samir A. A., Hiroaki O., Tetsuo I., Atsuko S., Ikuko M., Matsumi D., Munehiro N. (2001). Tetrahedron.

