# Correction: Swietemicrolides A–D, mexicanolide-type limonoids from the bark of one *Swietenia* species from Vietnam with *in vitro* cytotoxic and α-glucosidase inhibitory activities

**DOI:** 10.1039/d5ra90138c

**Published:** 2025-11-26

**Authors:** Tu-Quyen Thi Tran, Duong Hoang Trinh, Binh Thi Dieu Trinh, Dzung Ngoc Bui, Lien-Hoa Dieu Nguyen, Phuong Thu Tran

**Affiliations:** a Faculty of Chemistry, University of Science – Ho Chi Minh City – Vietnam 227 Nguyen Van Cu Street, District 5 Ho Chi Minh City 700000 Vietnam ttphuong@hcmus.edu.vn; b Vietnam National University – Ho Chi Minh City Linh Trung Ward, Thu Duc City Ho Chi Minh City 700000 Vietnam; c Institute of Drug Quality Control 200 Co Bac Street, District 1 Ho Chi Minh City 700000 Vietnam

## Abstract

Correction for ‘Swietemicrolides A–D, mexicanolide-type limonoids from the bark of one *Swietenia* species from Vietnam with *in vitro* cytotoxic and α-glucosidase inhibitory activities’ by Tu-Quyen Thi Tran *et al.*, *RSC Adv.*, 2024, **14**, 18608–18616, https://doi.org/10.1039/D4RA01954G.

The authors regret that the species name was incorrectly stated in the originally published article. The correct article title should be “Swietemicrolides A–D, mexicanolide-type limonoids from the bark of one *Swietenia* species from Vietnam with *in vitro* cytotoxic and α-glucosidase inhibitory activities”.

In addition, the sentence which reads “*Swietenia microphylla* is a new species of the genus *Swietenia*, differing from *Swietenia macrophylla* because of its white grey bark and smaller-sized leaves.^17,18^” should be updated to read “*Swietenia microphylla*, a species of *Swietenia*, was investigated in this work. In Vietnam and several other countries, including India, the species is referred to as *S. microphylla* to distinguish it from *S. macrophylla*, based on its white grey bark and smaller-sized leaves.^17,18^” to add clarity for readers.

The Royal Society of Chemistry apologises for these errors and any consequent inconvenience to authors and readers.

