# Supplementary material for: Swietemicrolides A–D, mexicanolide-type limonoids from the bark of Swietenia macrophylla with in vitro cytotoxic and α-glucosidase inhibitory activities
Source: RSC Adv. 2024 Jun 11;14(26):18608–16. doi: 10.1039/d4ra01954g (PMC11165692; doi:10.1039/d4ra01954g)
Supplement: RA-014-D4RA01954G-s001 [file RA-014-D4RA01954G-s001.pdf]

Electronic Supplementary Material (ESI) for RSC Advances.
This journal is © The Royal Society of Chemistry 2024

Supporting Information

Swietemicrolides A–D, mexicanolide-type limonoids from the bark of *Swietenia microphylla* with *in vitro* cytotoxic and *α*-glucosidase inhibitory activities

Tu-Quyen Thi Tran,[a,b] Duong Hoang Trinh,[c] Binh Thi Dieu Trinh,[a,b] Dzung Ngoc Bui,[a,b] Lien-Hoa Dieu Nguyen,[a,b] Phuong Thu Tran*[a,b]

[a]*Faculty of Chemistry, University of Science – Ho Chi Minh City – Vietnam, 227 Nguyen Van Cu Street, District 5, 700000 Ho Chi Minh City, Vietnam*

[b]*Vietnam National University – Ho Chi Minh City, Linh Trung Ward, Thu Duc City, 700000 Ho Chi Minh City, Vietnam*

[c]*Institute of Drug Quality Control, 200 Co Bac Street, District 1, 700000 Ho Chi Minh City, Vietnam*

Corresponding author:

*E-mail address: ttphuong@hcmus.edu.vn

Table of Contents

Table S1. ^{1}H (500 MHz) and ^{13}C (125 MHz) NMR data for swiemahogin A (**5**) in acetone-d_6....4

Table S2. ^{1}H (500 MHz) and ^{13}C (125 MHz) NMR data for *β*-sitosterol-3-*O*-*β*-D-glucopyranoside (**6**) pyridine-d_5....5

Table S3. ^{1}H (500 MHz) and ^{13}C (125 MHz) NMR data for homovanillyl alcohol (**7**) in acetone-d_6....7

Figure S1. UV spectrum of swetemicrolide A (**1**). 8

Figure S2. IR spectrum of swetemicrolide A (**1**). 8

Figure S3. HRESIMS spectrum of swetemicrolide A (**1**). 8

Figure S4. ^{1}H NMR spectrum of swetemicrolide A (**1**) in $CDCl_3$ (500MHz) 9

Figure S5. ^{13}C NMR spectrum of swetemicrolide A (**1**) in $CDCl_3$ (125MHz) 9

Figure S6. HSQC spectrum of swetemicrolide A (**1**) in $CDCl_3$ 10

Figure S7. HMBC spectrum of swetemicrolide A (**1**) in $CDCl_3$ 10

Figure S8. ^{1}H – ^{1}H COSY spectrum of swetemicrolide A (**1**) in $CDCl_3$ 11

Figure S9. NOESY spectrum of swetemicrolide A (**1**) in $CDCl_3$ 11

Figure S10. UV spectrum of swetemicrolide B (**2**). 12

Figure S11. IR spectrum of swetemicrolide B (**2**). 12

Figure S12. HRESIMS spectrum of swetemicrolide B (**2**). 12

Figure S13. ^{1}H NMR spectrum of swetemicrolide B (**2**) in $CDCl_3$ (500MHz) 13

Figure S14. ^{13}C NMR spectrum of swetemicrolide B (**2**) in $CDCl_3$ (125MHz) 13

Figure S15. HSQC spectrum of swetemicrolide B (**2**) in $CDCl_3$ 14

Figure S16. HMBC spectrum of swetemicrolide B (**2**) in $CDCl_3$ 14

Figure S17. ^{1}H – ^{1}H COSY spectrum of swetemicrolide B (**2**) in $CDCl_3$ 15

Figure S18. NOESY spectrum of swetemicrolide B (**2**) in $CDCl_3$ 15

Figure S19. UV spectrum of swetemicrolide C (**3**). 16

Figure S20. IR spectrum of swetemicrolide C (**3**). 16

Figure S21. HRESIMS spectrum of swetemicrolide C (**3**). 16

Figure S22. ^{1}H NMR spectrum of swetemicrolide C (**3**) in $CDCl_3$ (500MHz) 17

Figure S23. ^{13}C NMR spectrum of swetemicrolide C (**3**) in $CDCl_3$ (125MHz) 17

Figure S24. HSQC spectrum of swetemicrolide C (**3**) in $CDCl_3$ 18

Figure S25. HMBC spectrum of swetemicrolide C (**3**) in $CDCl_3$ 18

Figure S26. ^1H – ^1H COSY spectrum of swetemicrolide C (**3**) in $CDCl_3$ 19
Figure S27. NOESY spectrum of swetemicrolide C (**3**) in $CDCl_3$ 19
Figure S28. UV spectrum of swetemicrolide D (**4**). 20
Figure S29. IR spectrum of swetemicrolide D (**4**). 20
Figure S30. HRESIMS spectrum of swetemicrolide D (**4**). 20
Figure S31. ^1H NMR spectrum of swetemicrolide D (**4**) in $CDCl_3$ (500MHz) 21
Figure S32. ^{13}C NMR spectrum of swetemicrolide D (**4**) in $CDCl_3$ (125MHz) 21
Figure S33. HSQC spectrum of swetemicrolide D (**4**) in $CDCl_3$ 22
Figure S34. HMBC spectrum of swetemicrolide D (**4**) in $CDCl_3$ 22
Figure S35. ^1H – ^1H COSY spectrum of swetemicrolide D (**4**) in $CDCl_3$ 23
Figure S36. NOESY spectrum of swetemicrolide D (**4**) in $CDCl_3$ 23
Figure S37. ^1H NMR spectrum of swiemahogin A (**5**) in acetone-d_6 (500MHz) 24
Figure S38. ^{13}C NMR spectrum of swiemahogin A (**5**) in acetone-d_6 (125MHz) 24
Figure S39. ^1H NMR spectrum of *β*-sitosterol-3-*O*-*β*-D-glucopyranoside (**6**) in pyridine-d_5 (500MHz) 25
Figure S40. ^{13}C NMR spectrum of *β*-sitosterol-3-*O*-*β*-D-glucopyranoside (**6**) in pyridine-d_5 (125MHz) 25
Figure S41. ^1H NMR spectrum of homovanillyl alcohol (**7**) in acetone-d_6 (500MHz) 26
Figure S42. ^{13}C NMR spectrum of homovanillyl alcohol (**7**) in acetone-d_6 (125MHz) ...26

Table S1. ^{1}H (500 MHz) and ^{13}C (125 MHz) NMR data for swiemahogin A (5) in acetone-d_6

No.	δ_H (J in Hz)	δ_C
1	7.06 d (10.5)	154.9
2	5.86 d (10.5)	125.8
3	-	203.0
4	-	46.9
5	2.64 dd (6.1, 3.1)	45.6
6	H_a: 2.58 dd (17.0, 6.2)<br>H_b: 2.42 dd (17.0, 3.1)	33.1
7	-	175.6
8	-	93.5
9	1.81 m	52.5
10	-	45.0
11	1.56 m	22.3
12	1.51 m; 1.30 m	34.5
13	-	43.0
14	-	87.3
15	2.95 d (16.8); 2.30 d (16.8)	43.2
16	-	174.5
17	5.47 m	71.4
18	1.08 s	17.8
19	1.41 s	20.2
20	-	126.6
21	7.57 brs	142.0
22	6.49 brs	111.3
23	7.51 t (1.4)	143.8
28	1.00 s	24.0
29	1.11 s	23.3
30	1.90 s	16.7
7-OCH_3	3.67 s	52.8
14-OH	6.77 dd (5.2, 2.3)	
17-OH	5.65 t (3.2)	

Table S2. ^{1}H (500 MHz) and ^{13}C (125 MHz) NMR data for *β*-sitosterol-3-*O*-*β*-D-glucopyranoside (6) in pyridine-d_5

No.	δ_H (J in Hz)	δ_C
1		38.0
2		30.8
3	4.00 m	78.7
4		39.9
5		141.5
6	5.38 m	122.4
7		32.7
8		32.6
9		50.9
10		37.5
11		21.8
12		40.5
13		43.0
14		57.4
15		25.0
16		29.1
17		56.8
18	0.69 s	12.5
19	0.96 s	19.8
20		36.9
21	1.01 d (6.5)	19.6
22		34.8
23		27.0
24		46.6
25		30.0
26	0.93 d (7.4)	19.9
27	0.89 d (6.9)	20.5

28		24.0
29	0.91 t (6.9)	12.7
1′	5.08 d (7.8)	103.1
2′	4.08 t (8.2)	75.9
3′	4.31 m	79.1
4′	4.31 m	72.3
5′	4.00 m	79.0
6′	H_a: 4.59 dd (11.8, 2.5)<br>H_b: 4.44 dd (11.8, 5.3)	63.4

Table S3. ^{1}**H (500 MHz) and** ^{13}**C (125 MHz) NMR data for homovanillyl alcohol (7) in acetone-*d*$_6$**

No.	δ_H (J in Hz)	δ_C
1		145.8
2		148.3
3	6.83 d (1.4)	113.5
4		131.8
5	6.62 dd (8.0 và 1.5)	122.3
6	6.72 d (8.0)	115.6
1′	2.71 t (7.1)	40.0
2′	3.69 m	64.3
2-OCH_3	3.82 s	56.3
1-OH	7.29 s	
2′-OH	3.58 t (5.5)	

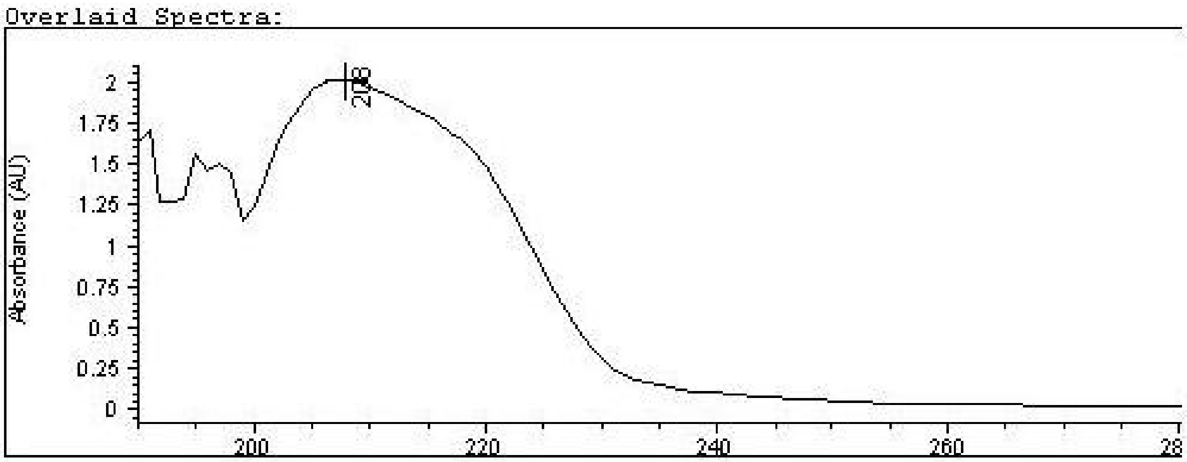

Figure S1. UV spectrum of swetemicrolide A (**1**)

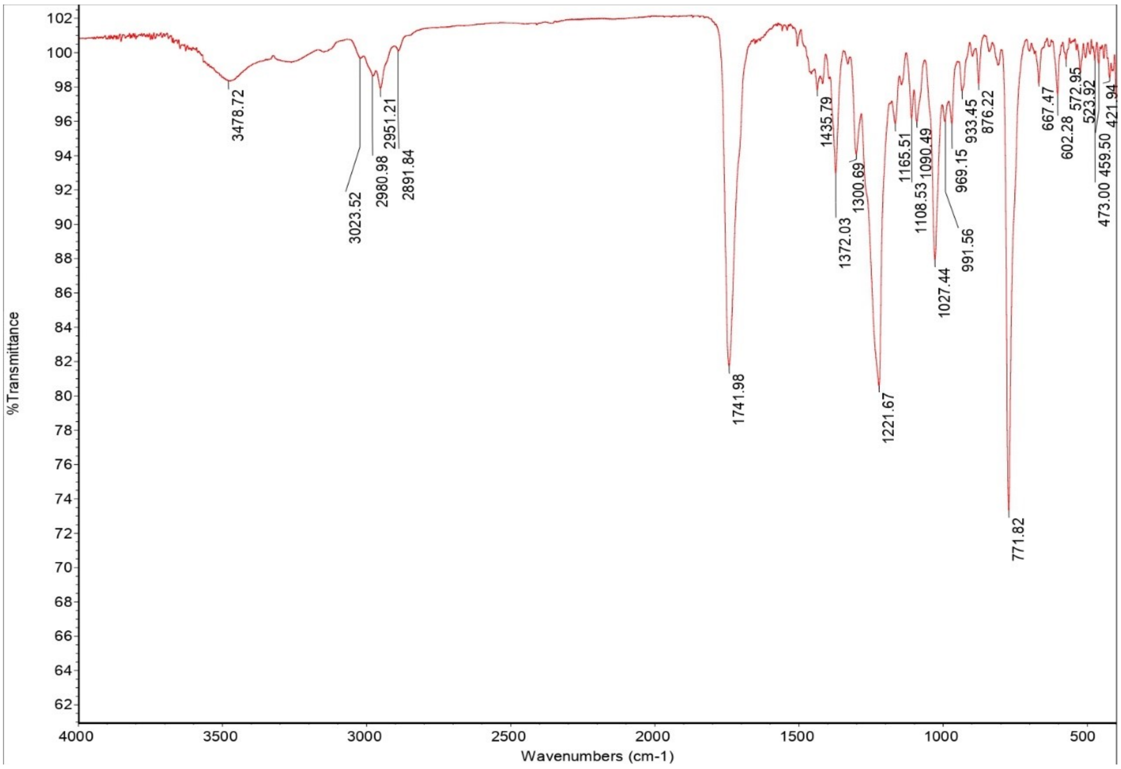

Figure S2. IR spectrum of swetemicrolide A (**1**)

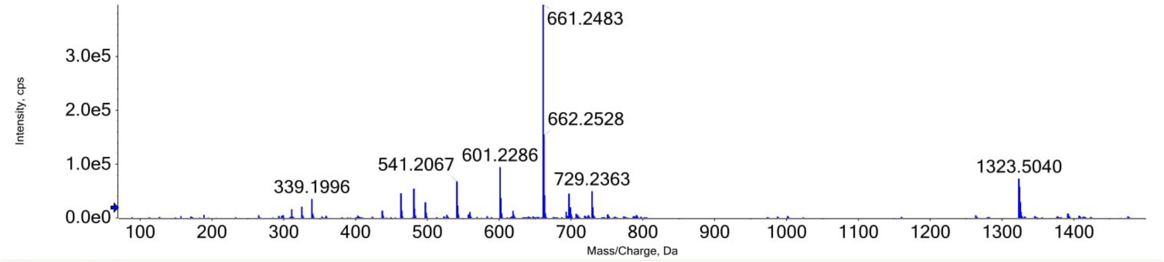

Figure S3. HRESIMS spectrum of swetemicrolide A (**1**)

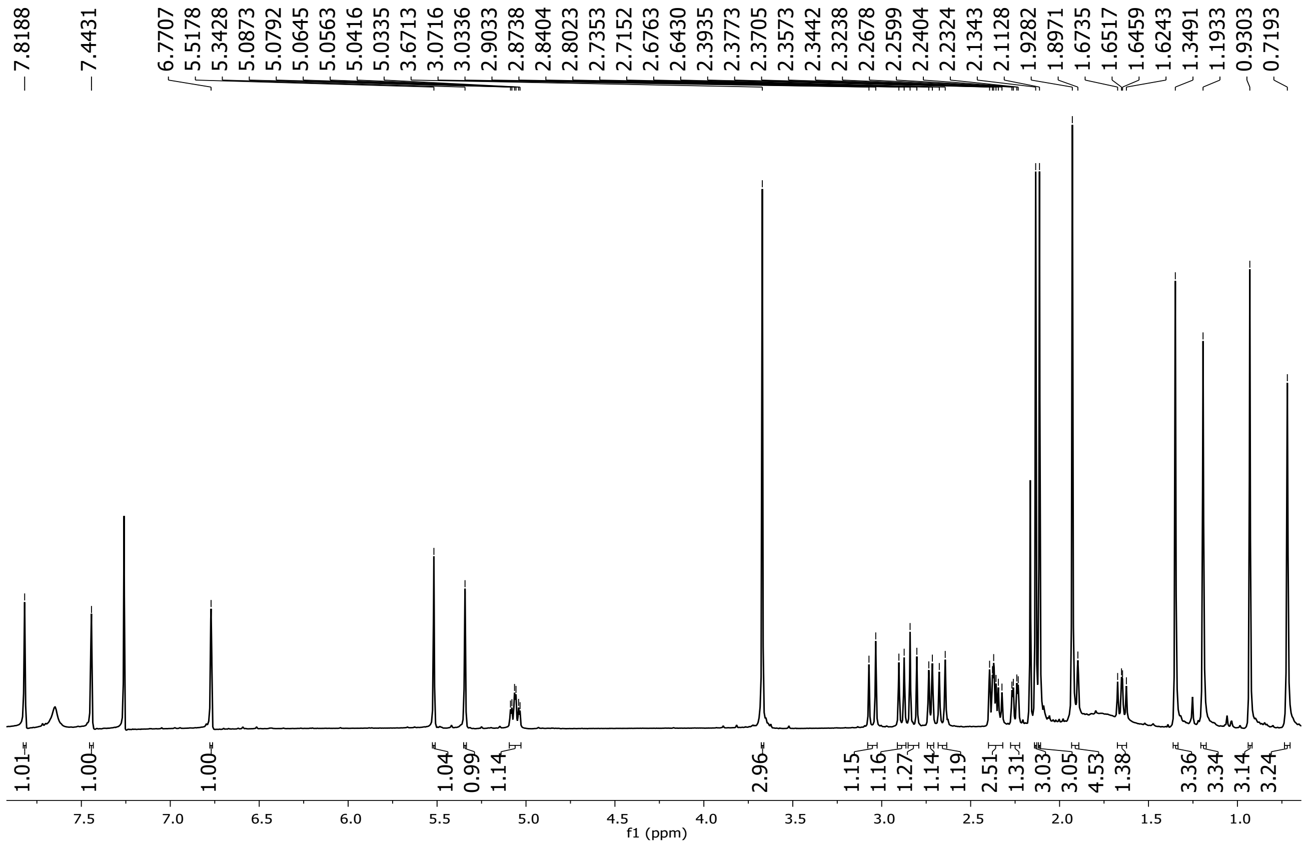

Figure S4. ^{1}H NMR spectrum of swetemicrolide A (**1**) in $CDCl_3$ (500MHz)

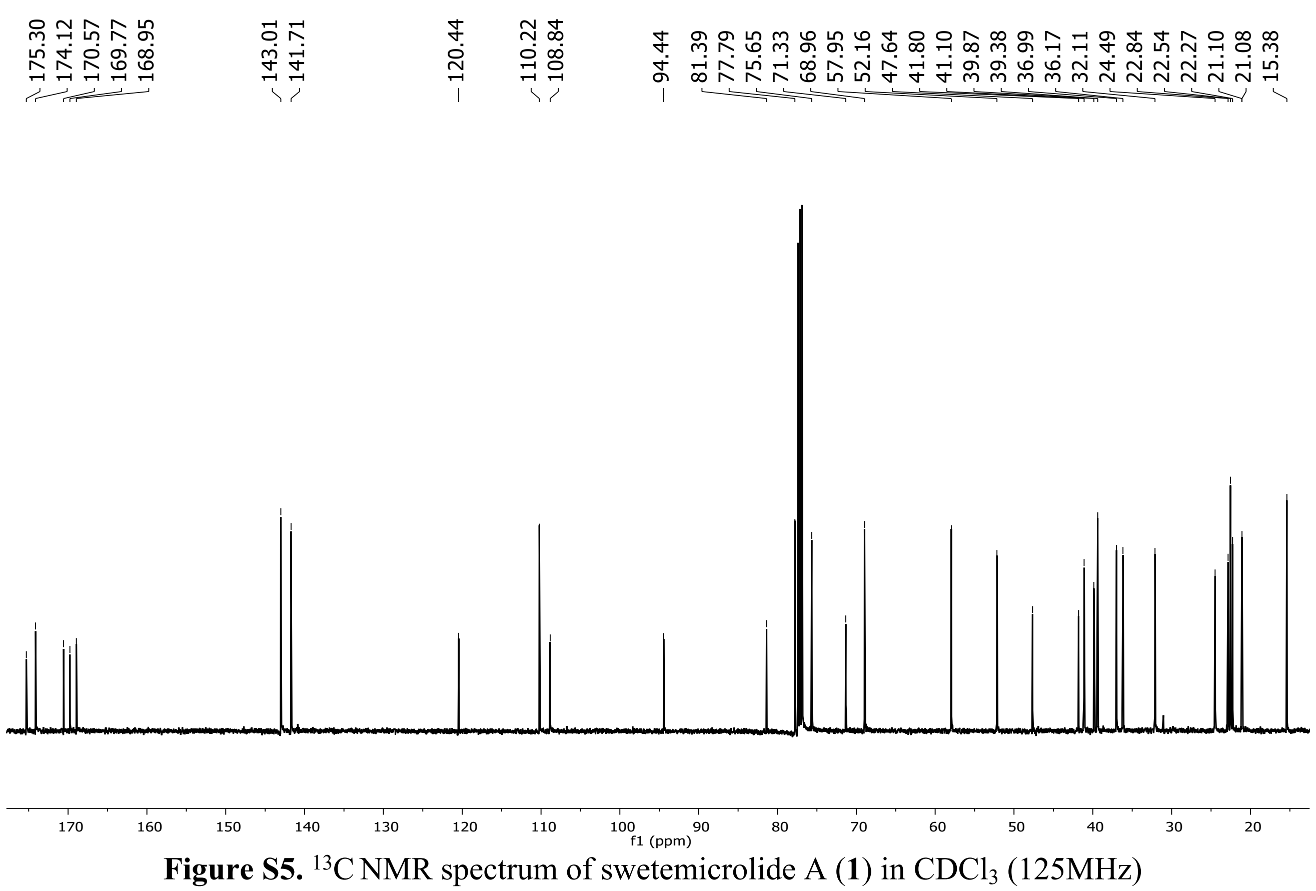

Figure S5. ^{13}C NMR spectrum of swetemicrolide A (**1**) in $CDCl_3$ (125MHz)

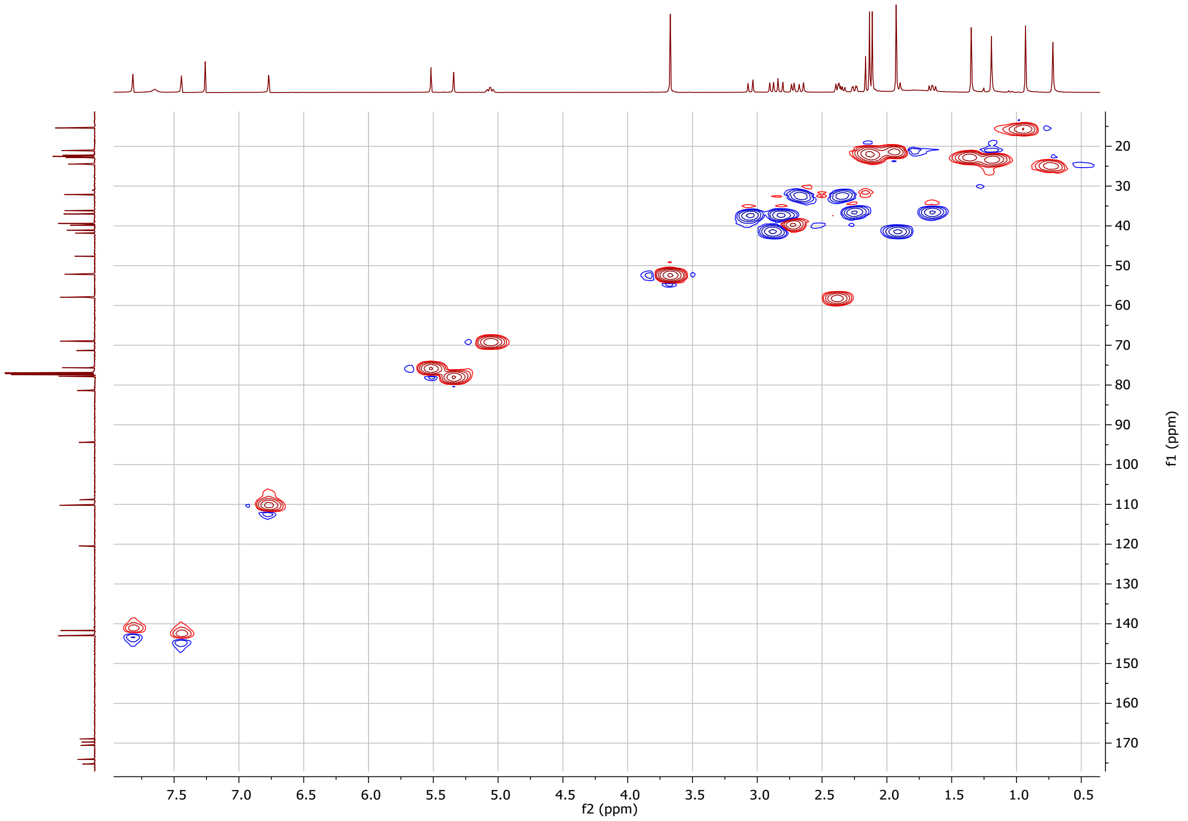

Figure S6. HMBC spectrum of swetemicrolide A (**1**) in $CDCl_3$

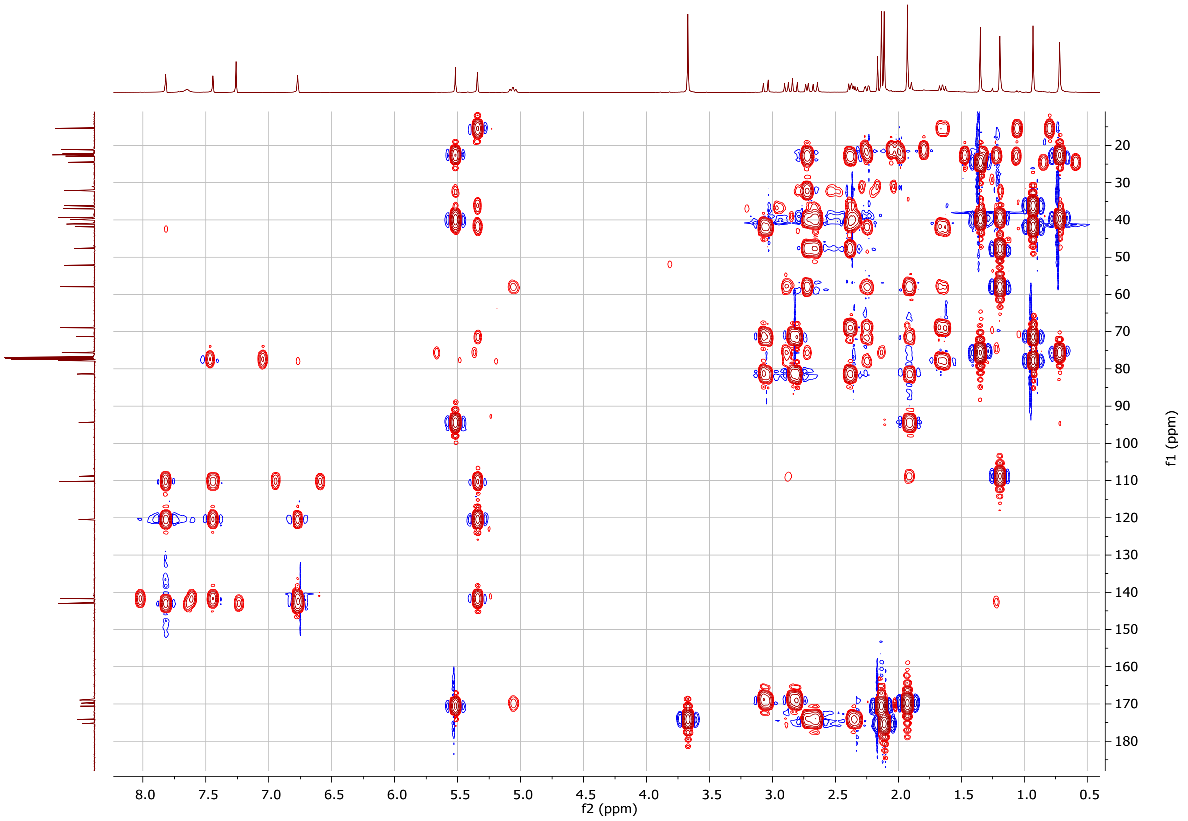

Figure S7. HMBC spectrum of swetemicrolide A (**1**) in $CDCl_3$

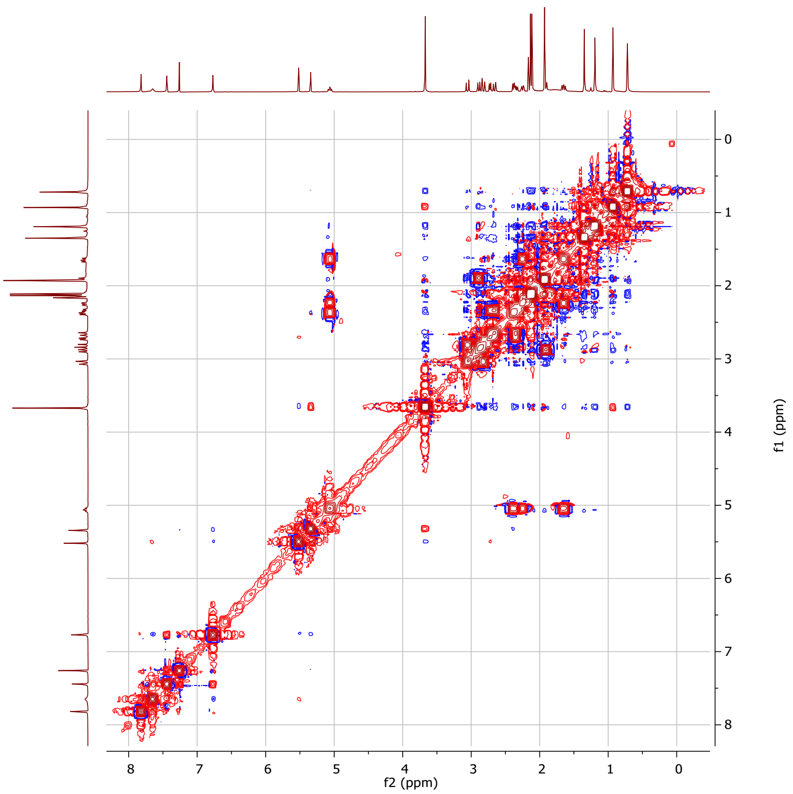

Figure S8. $^{1}H - ^{1}H$ COSY spectrum of swetemicrolide A (**1**) in $CDCl_3$

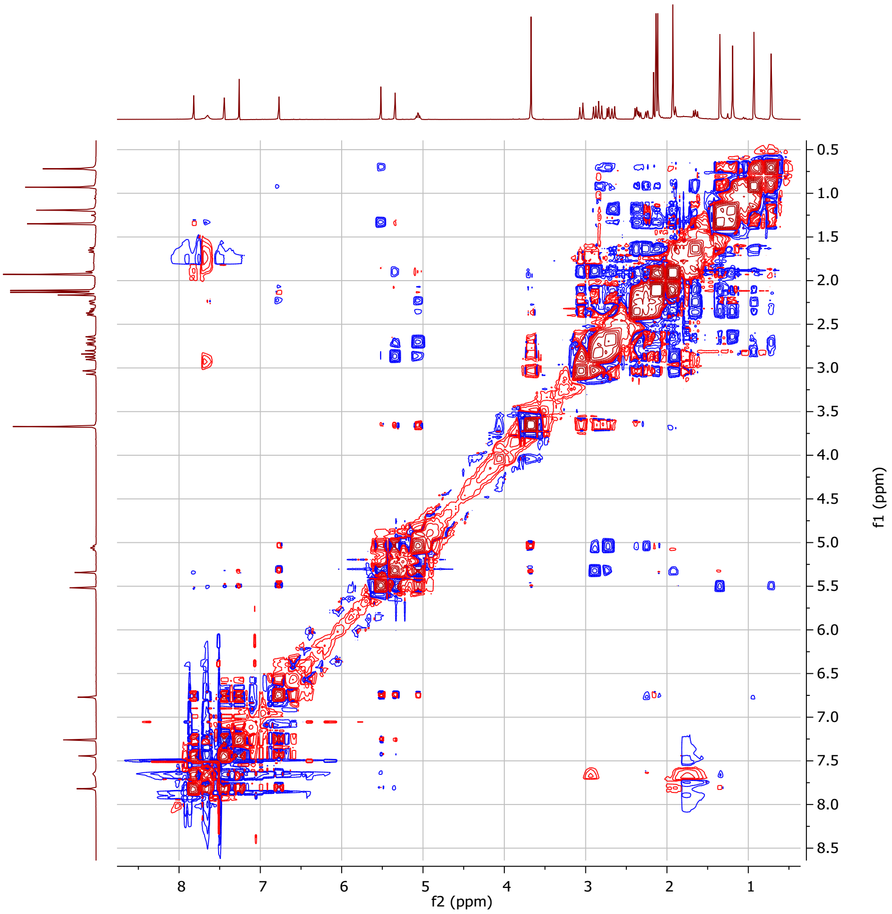

Figure S9. NOESY spectrum of swetemicrolide A (**1**) in $CDCl_3$

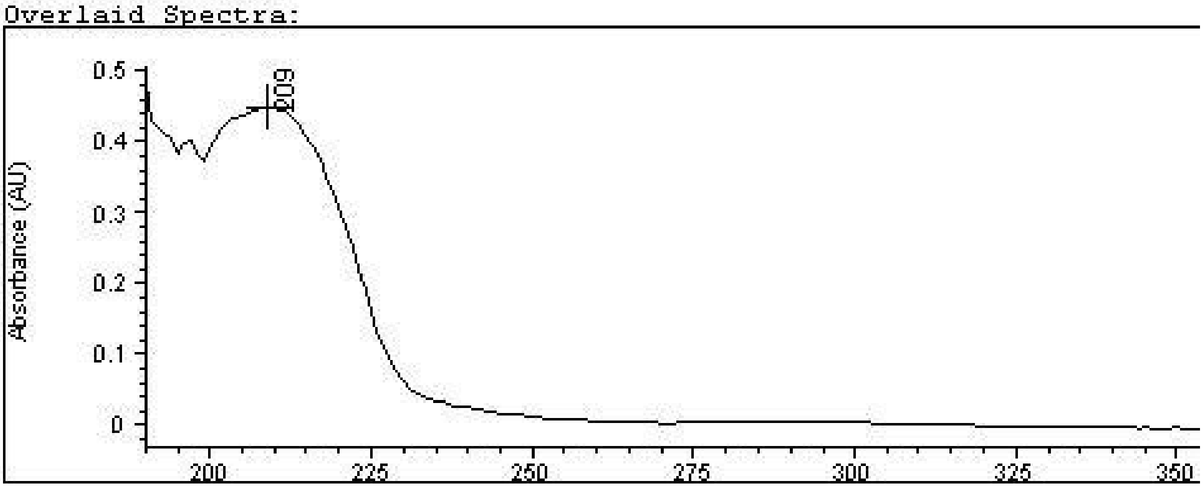

Figure S10. UV spectrum of swetemicrolide B (**2**).

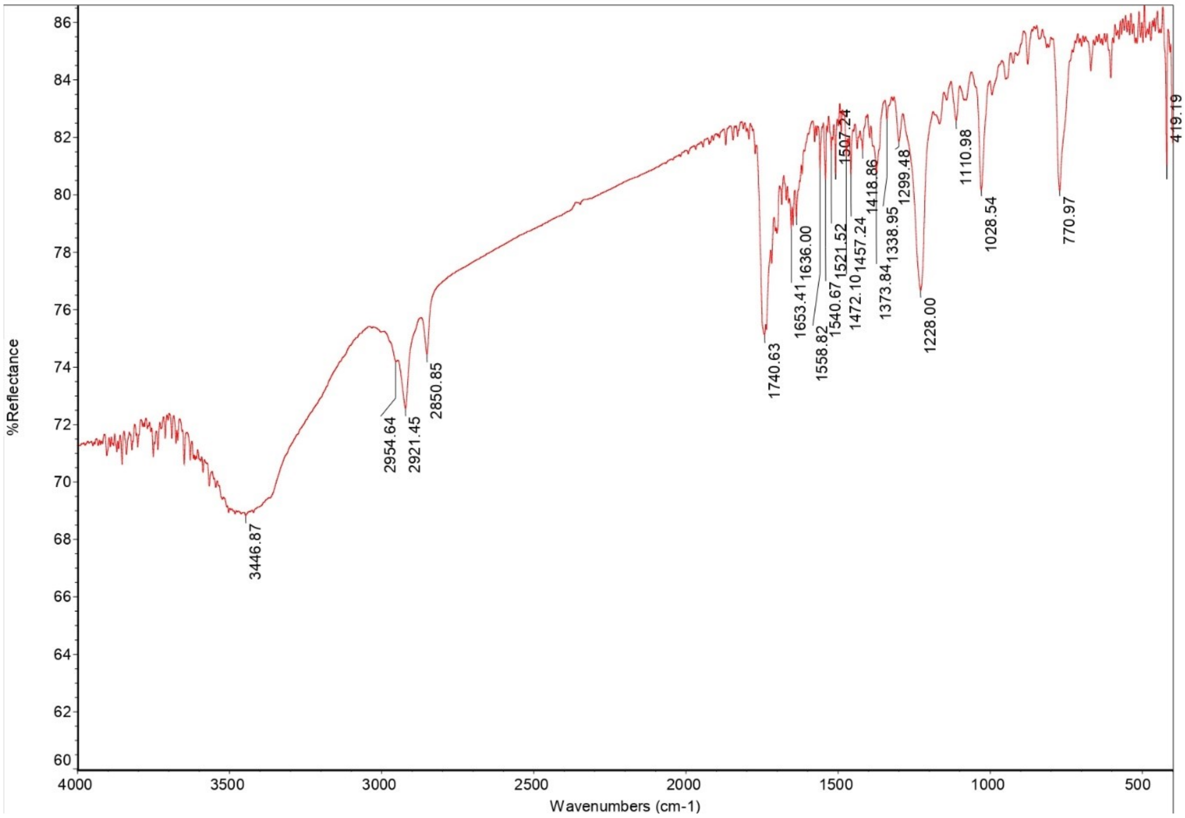

Figure S11. IR spectrum of swetemicrolide B (**2**).

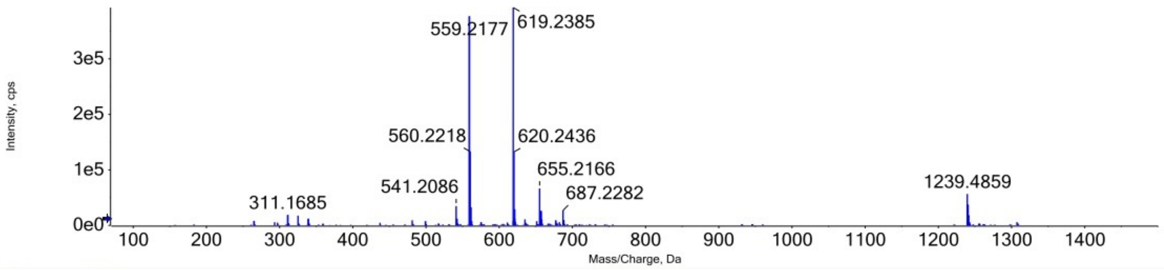

Figure S12. HRESIMS spectrum of swetemicrolide B (**2**).

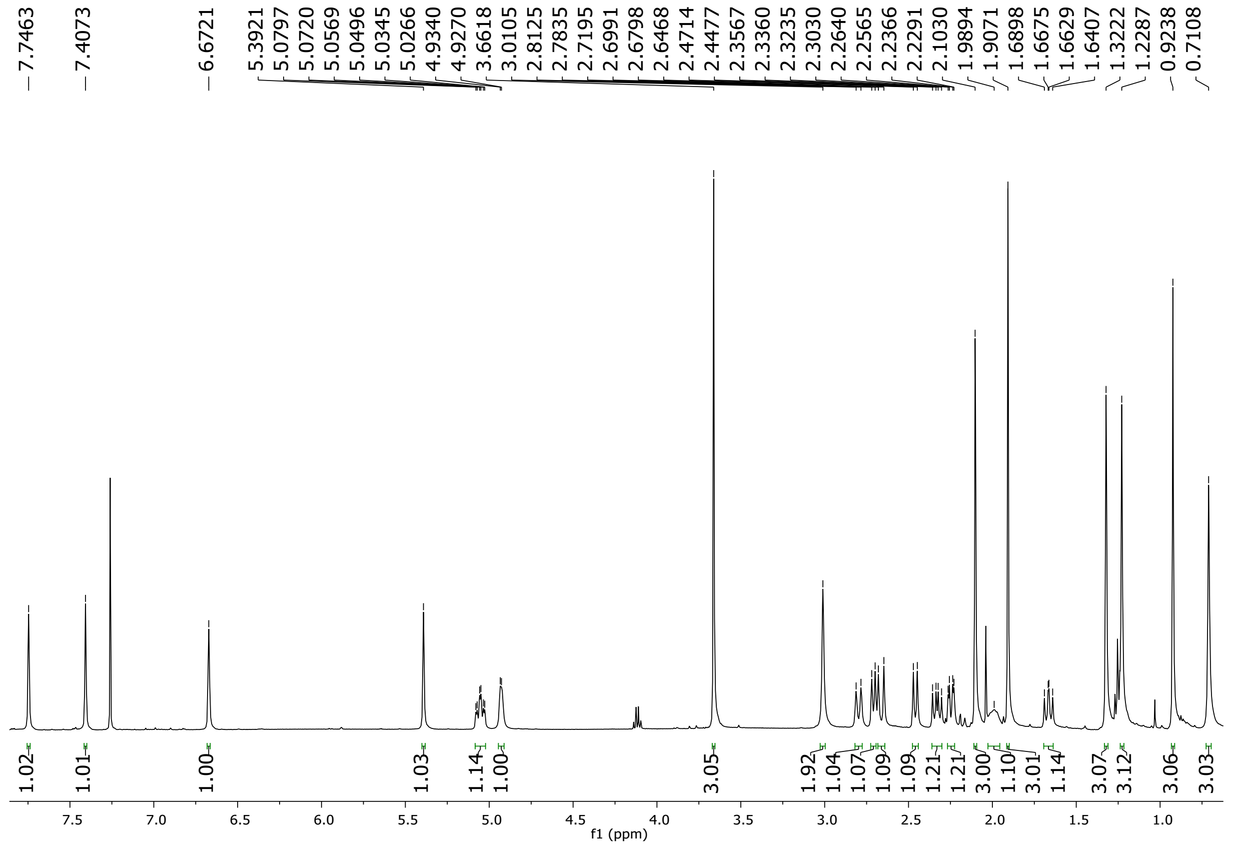

Figure S13. ^{1}H NMR spectrum of swetemicrolide B (**2**) in $CDCl_3$ (500MHz)

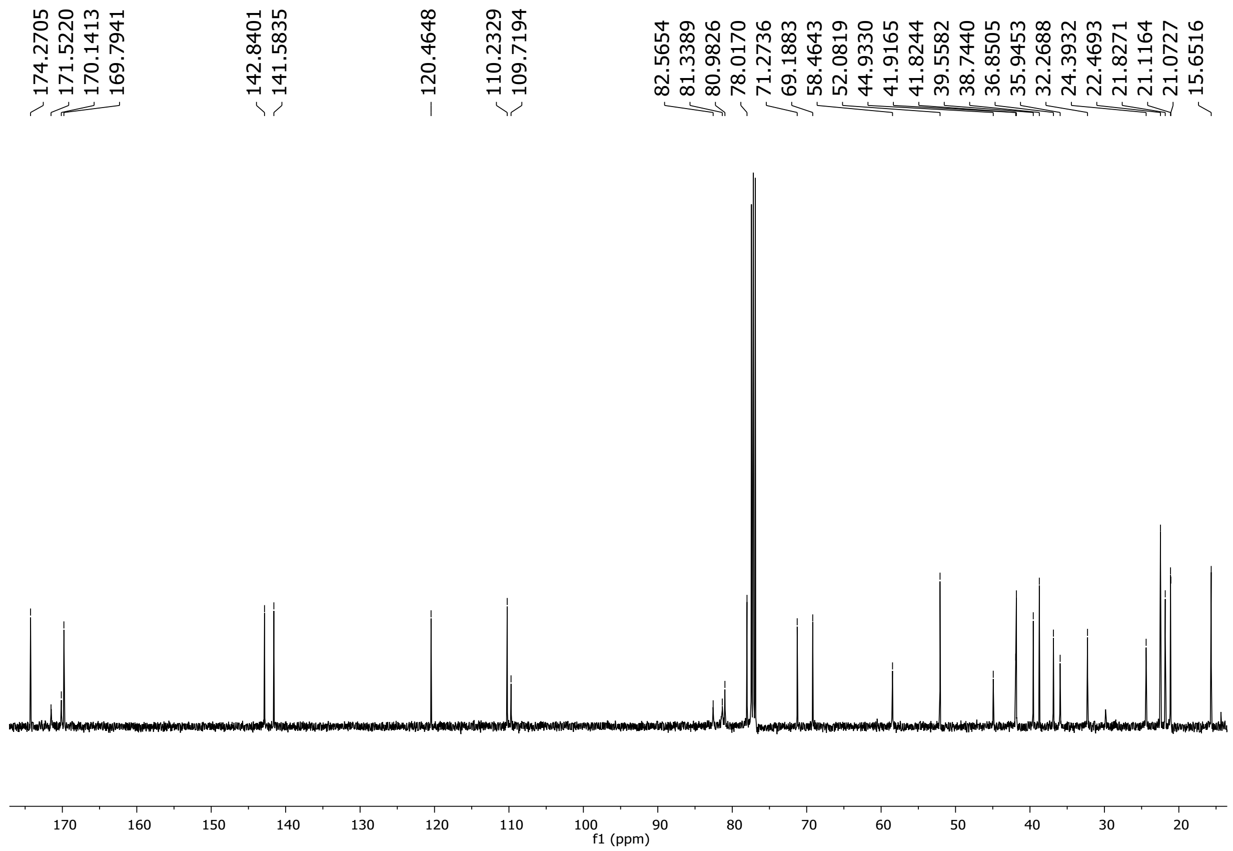

Figure S14. ^{13}C NMR spectrum of swetemicrolide B (**2**) in $CDCl_3$ (125MHz)

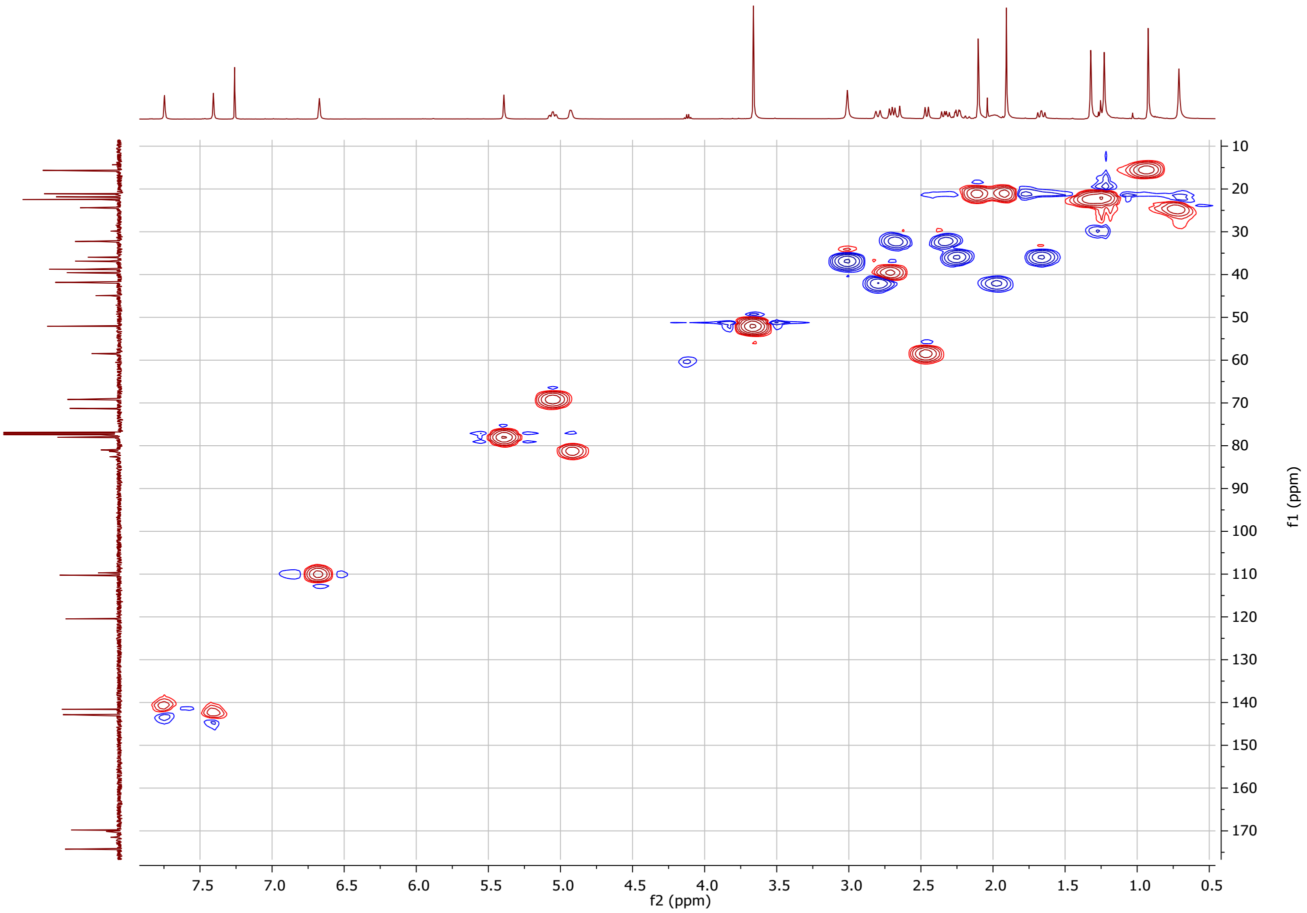

Figure S15. HSQC spectrum of swetemicrolide B (**2**) in $CDCl_3$

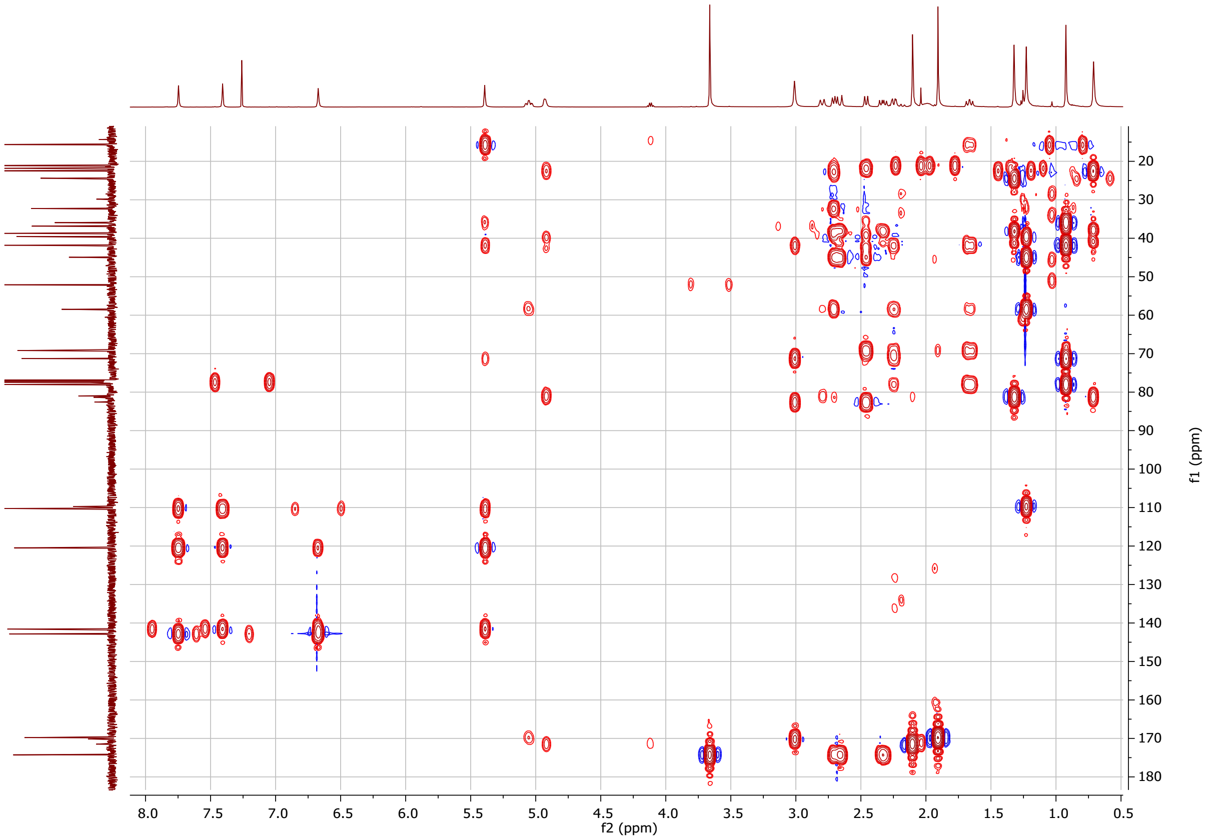

Figure S16. HMBC spectrum of swetemicrolide B (**2**) in $CDCl_3$

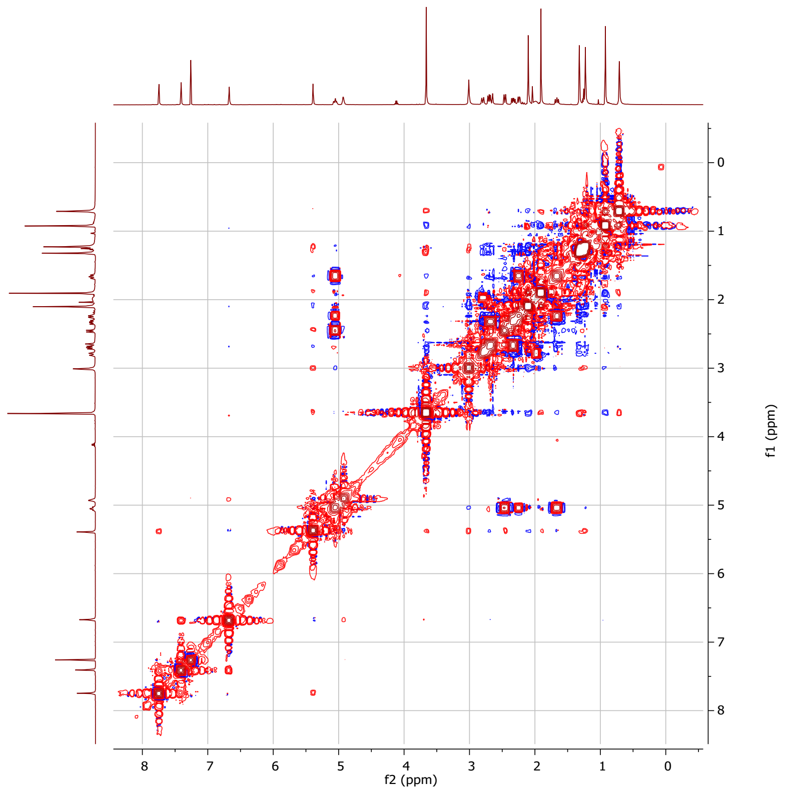

Figure S17. ${}^{1}H$ – ${}^{1}H$ COSY spectrum of swetemicrolide B (**2**) in $CDCl_3$

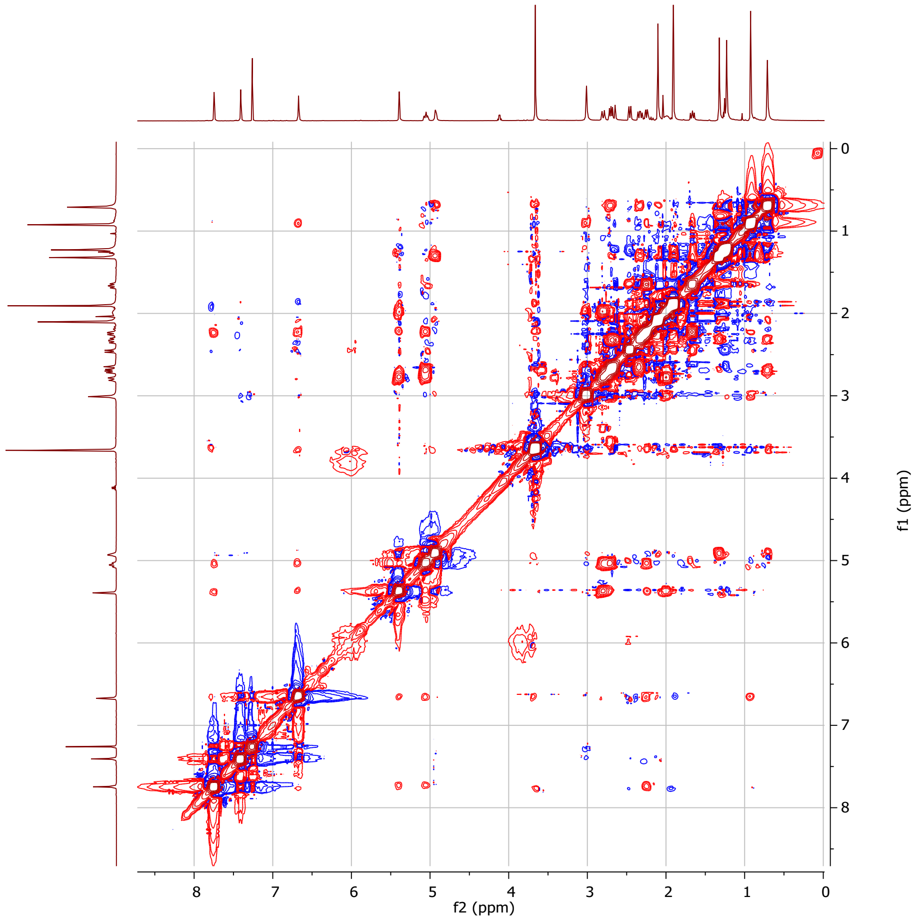

Figure S18. NOESY spectrum of swetemicrolide B (**2**) in $CDCl_3$

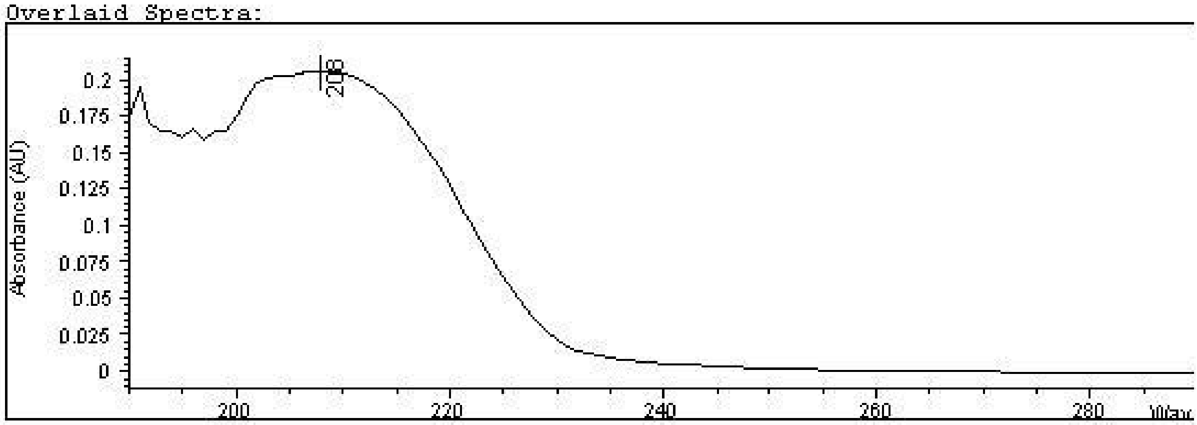

Figure S19. UV spectrum of swetemicrolide C (**3**).

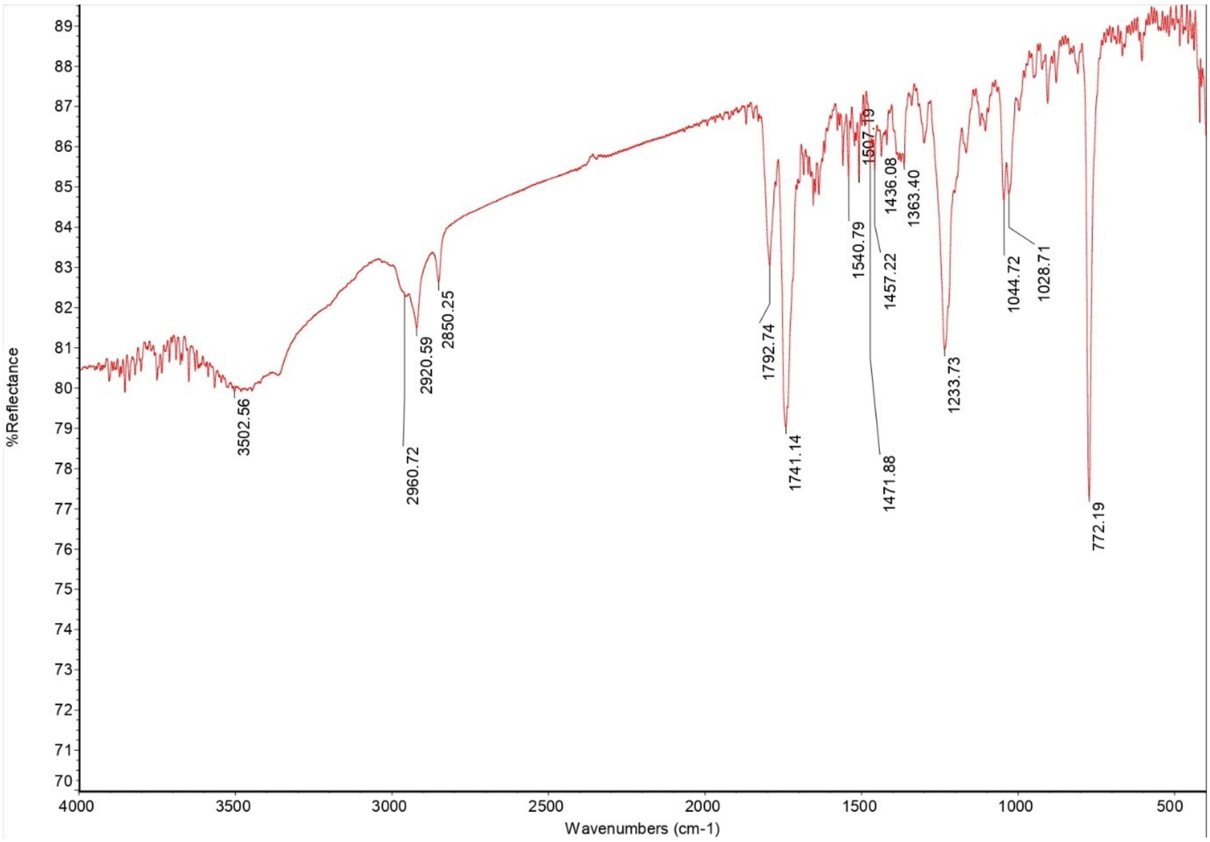

Figure S20. IR spectrum of swetemicrolide C (**3**).

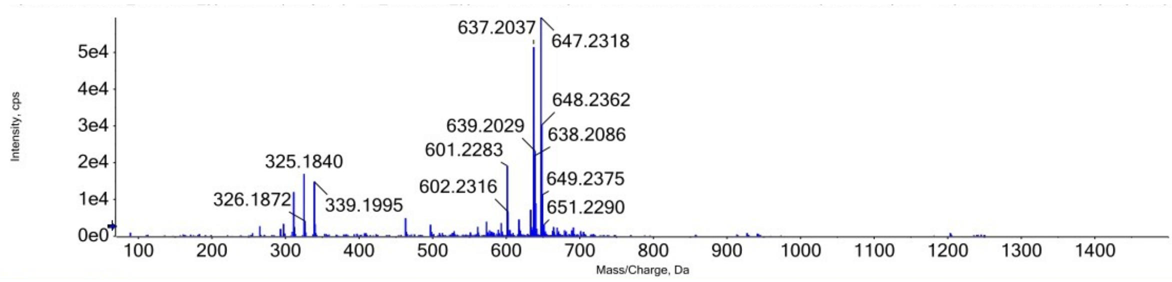

Figure S21. HRESIMS spectrum of swetemicrolide C (**3**).

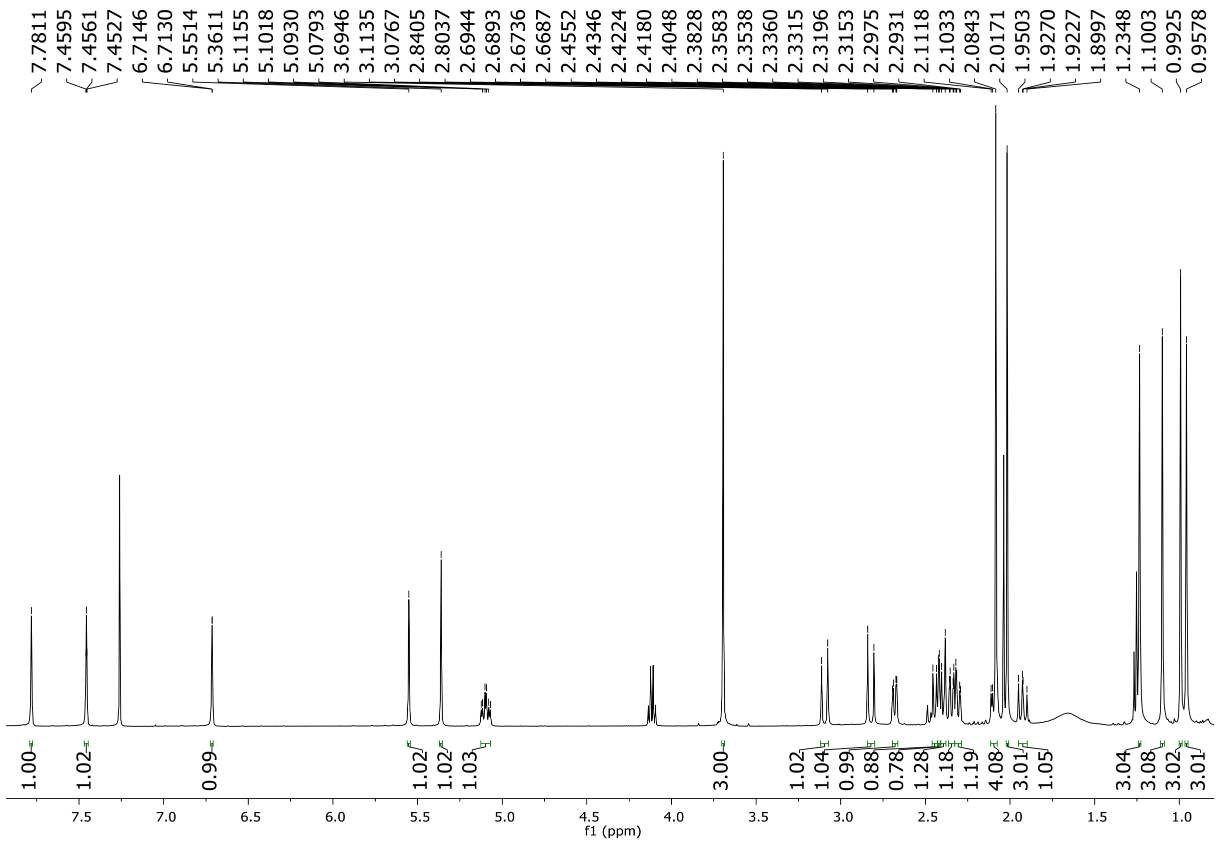

Figure S22. ^{1}H NMR spectrum of swetemicrolide C (**3**) in $CDCl_3$ (500MHz)

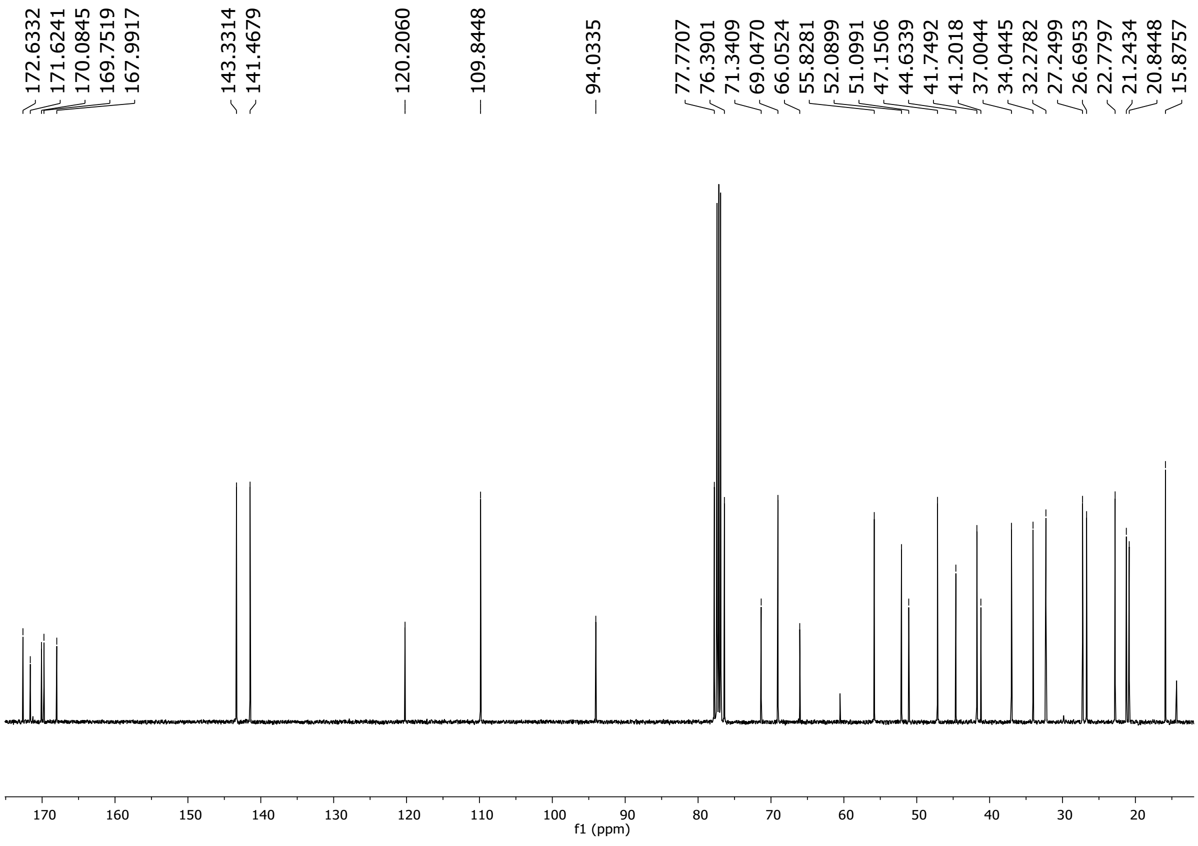

Figure S23. ^{13}C NMR spectrum of swetemicrolide C (**3**) in $CDCl_3$ (125MHz)

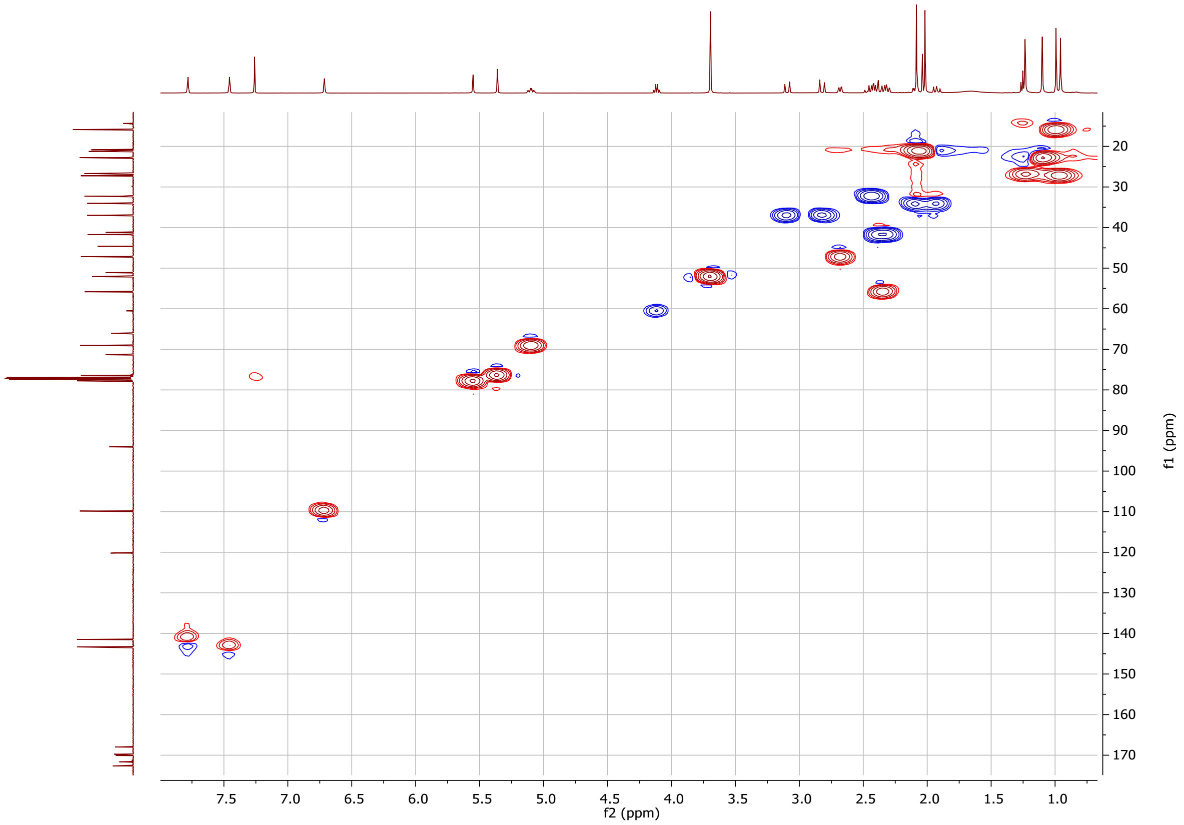

Figure S24. HSQC spectrum of swetemicrolide C (**3**) in $CDCl_3$

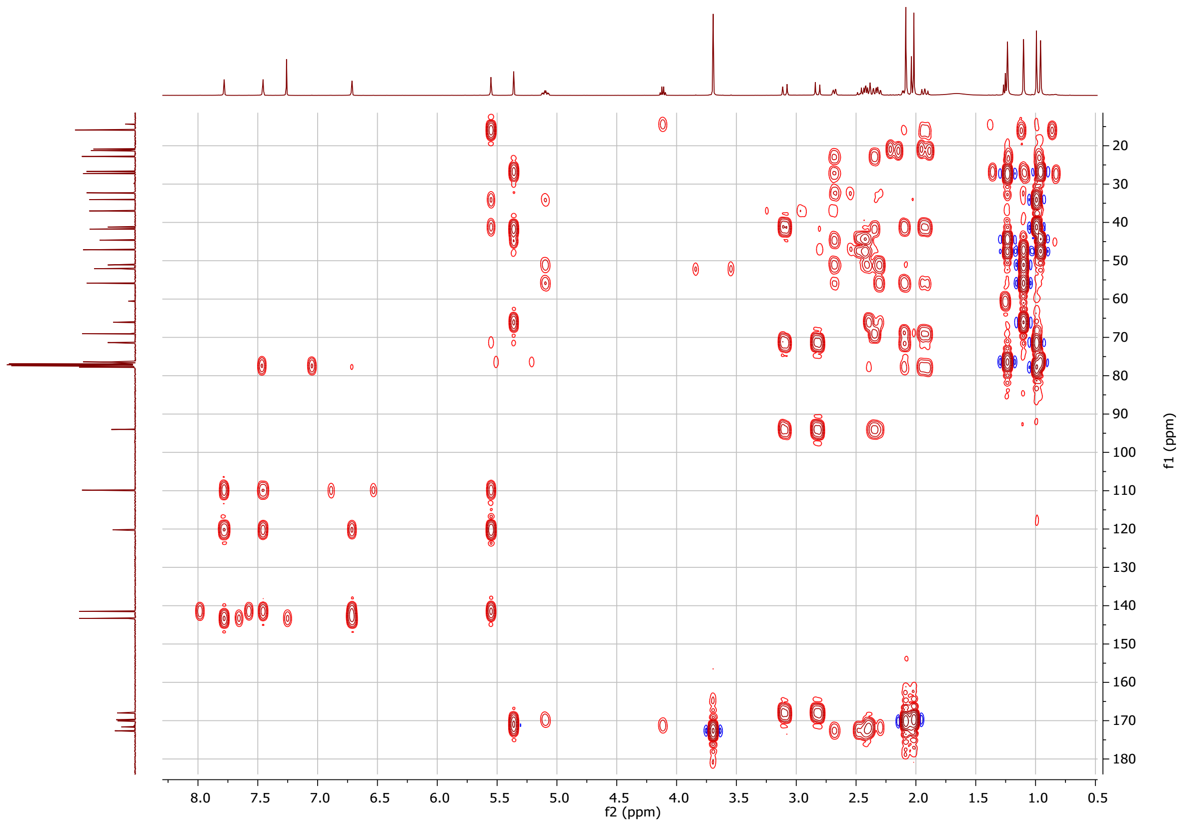

Figure S25. HMBC spectrum of swetemicrolide C (**3**) in $CDCl_3$

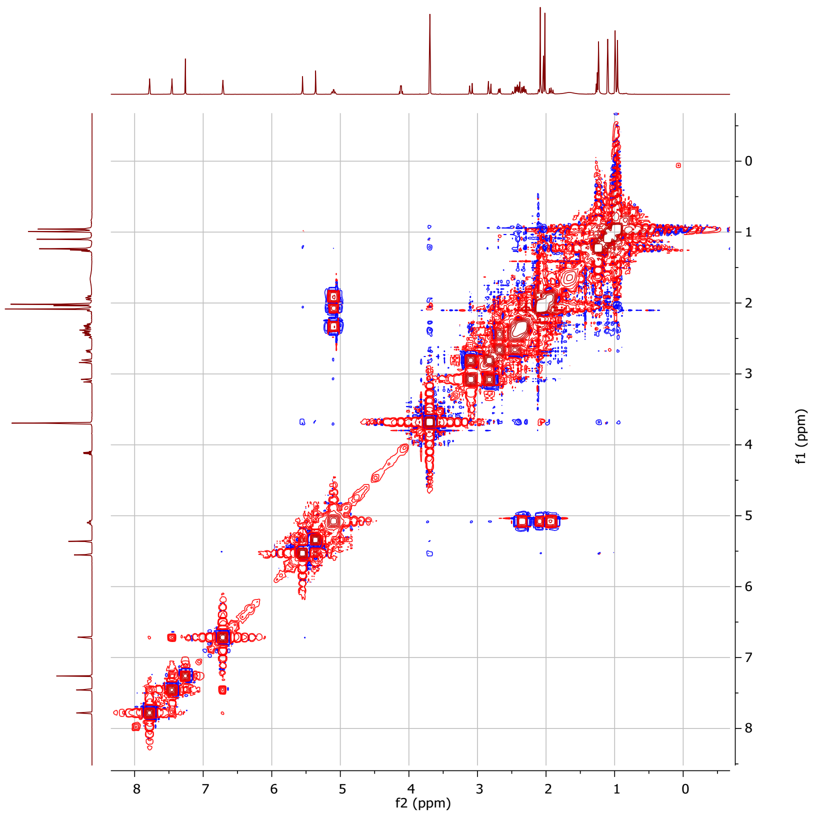

Figure S26. $^{1}H - ^{1}H$ COSY spectrum of swetemicrolide C (**3**) in $CDCl_3$

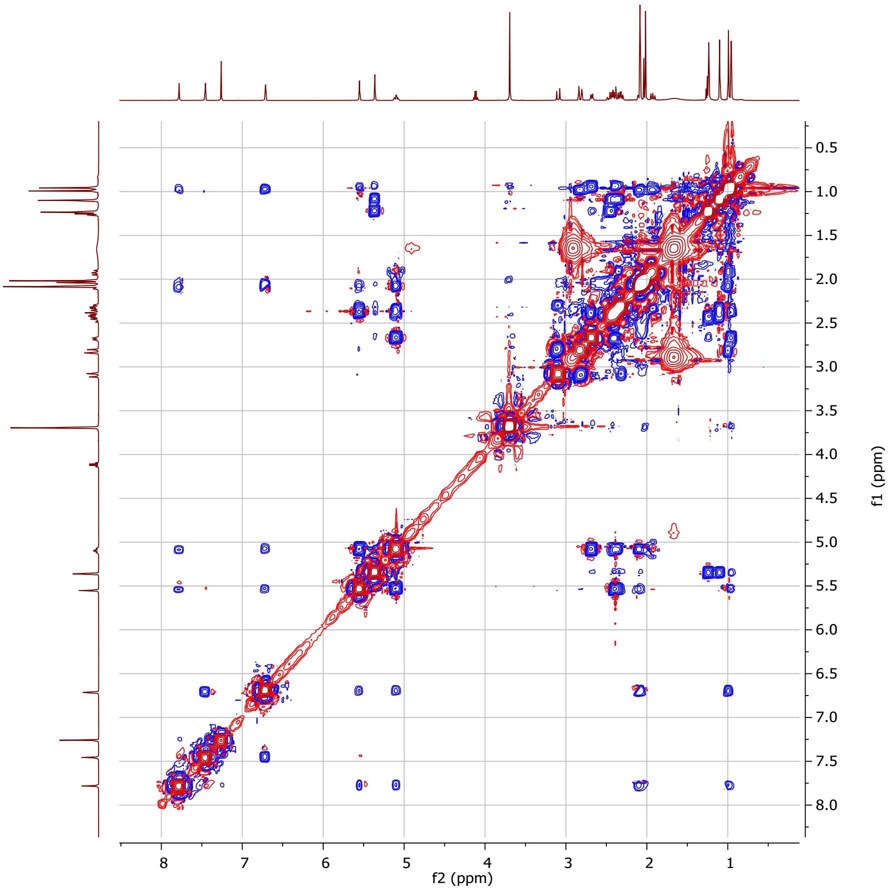

Figure S27. NOESY spectrum of swetemicrolide C (**3**) in $CDCl_3$

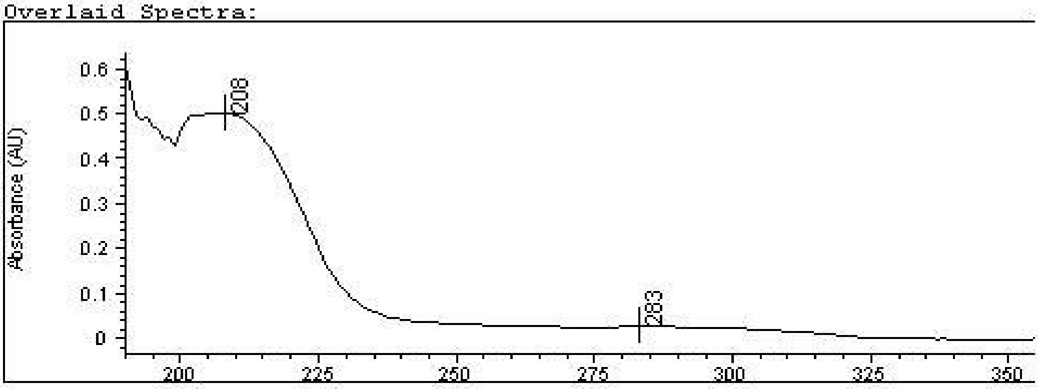

Figure S28. UV spectrum of swetemicrolide D (**4**).

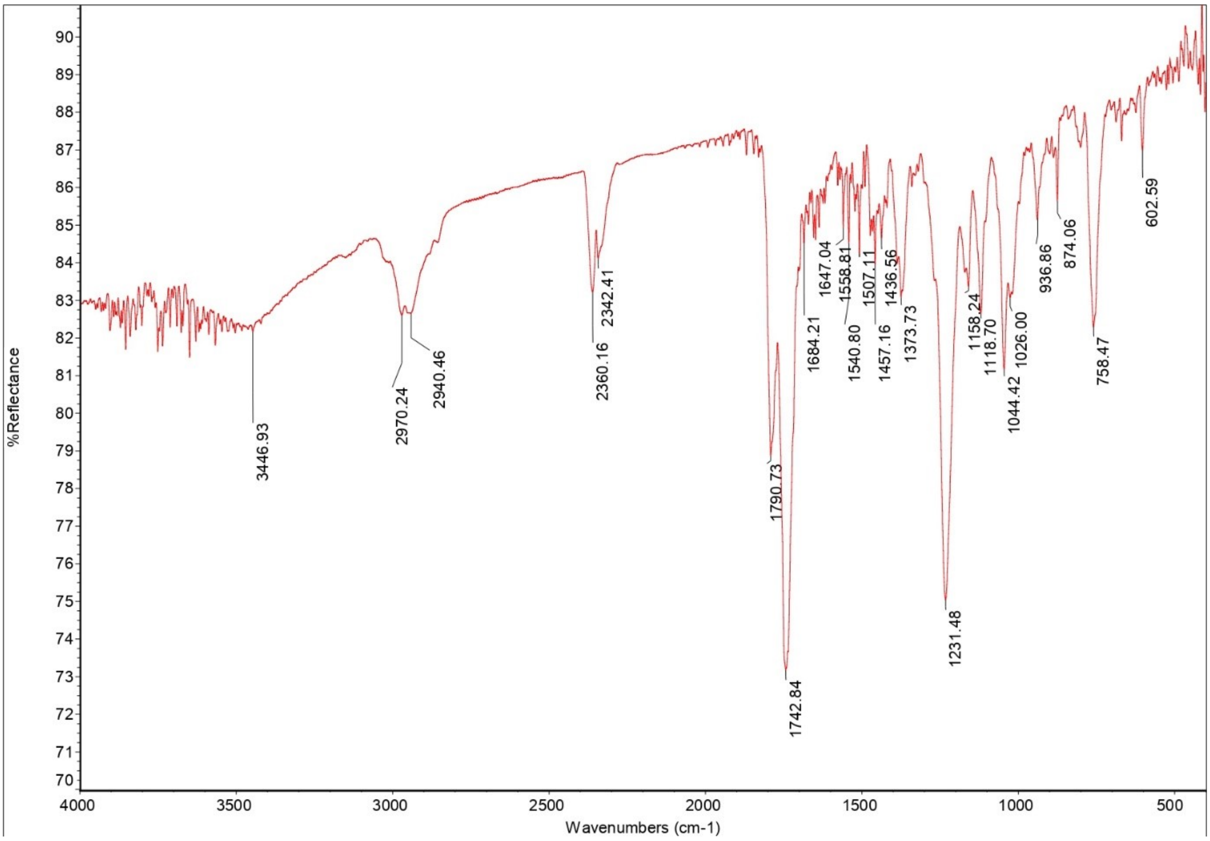

Figure S29. IR spectrum of swetemicrolide D (**4**).

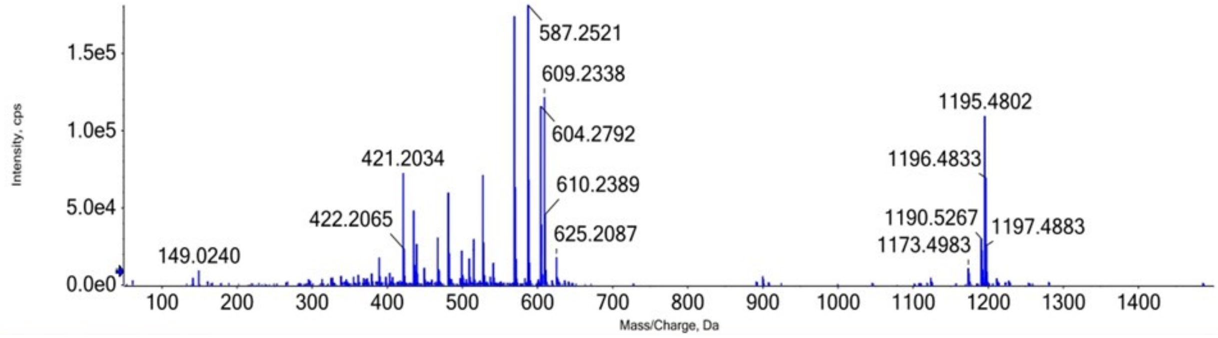

Figure S30. HRESIMS spectrum of swetemicrolide D (**4**).

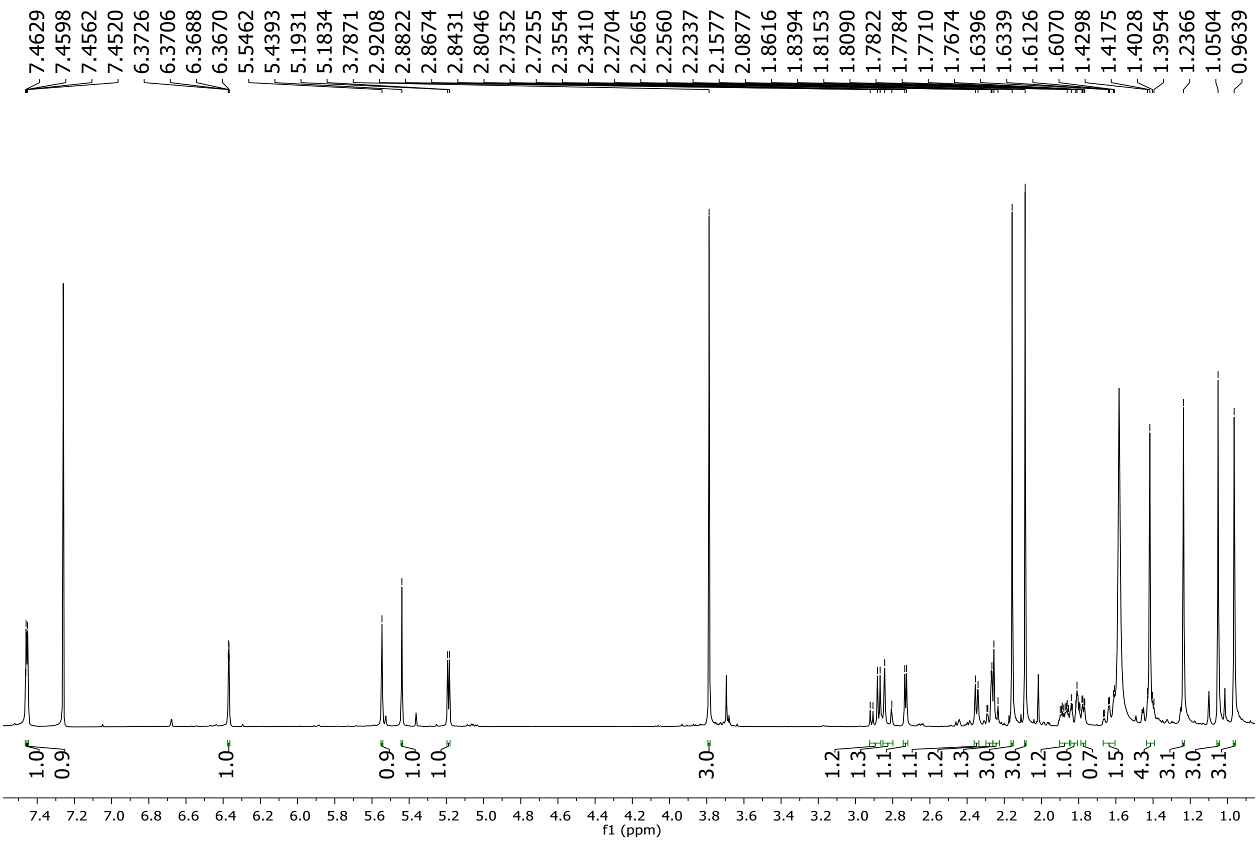

Figure S31. ^{1}H NMR spectrum of swetemicrolide D (**4**) in $CDCl_3$ (500MHz)

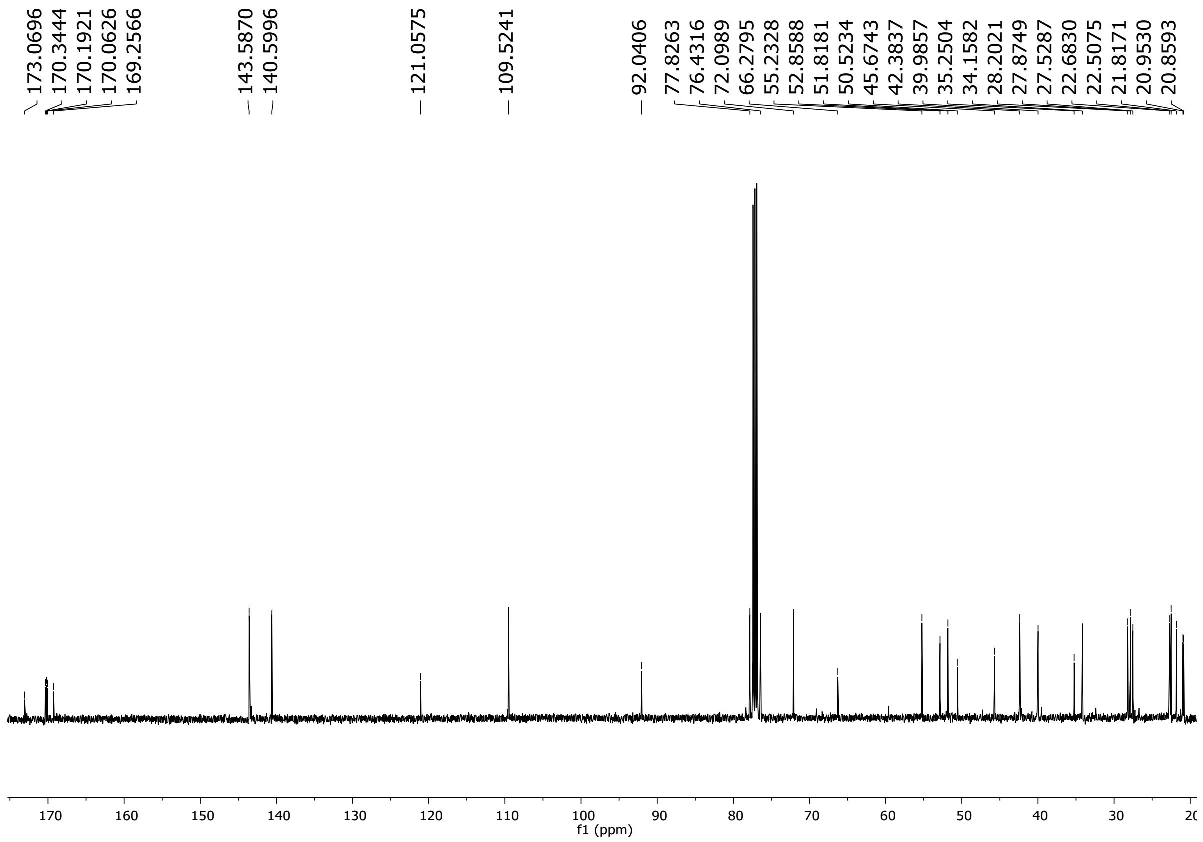

Figure S32. ^{13}C NMR spectrum of swetemicrolide D (**4**) in $CDCl_3$ (125MHz)

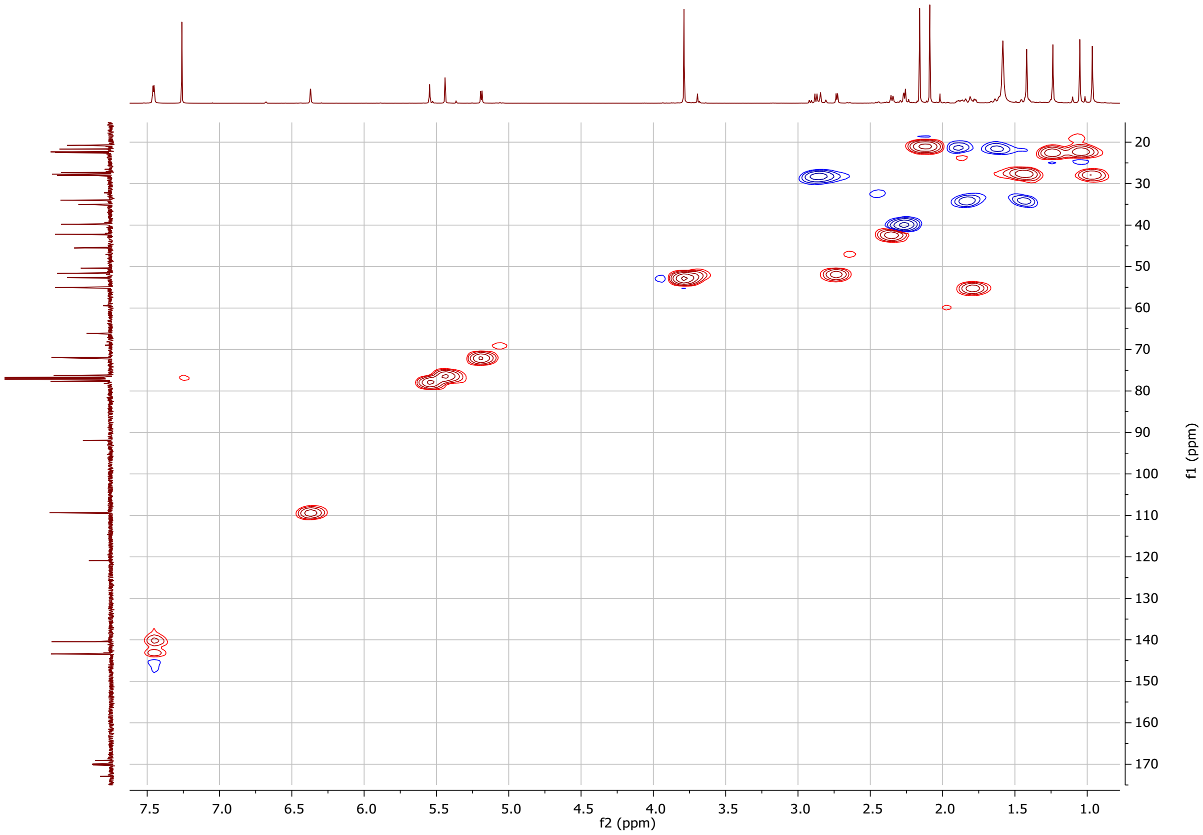

Figure S33. HSQC spectrum of swetemicrolide D (**4**) in $CDCl_3$

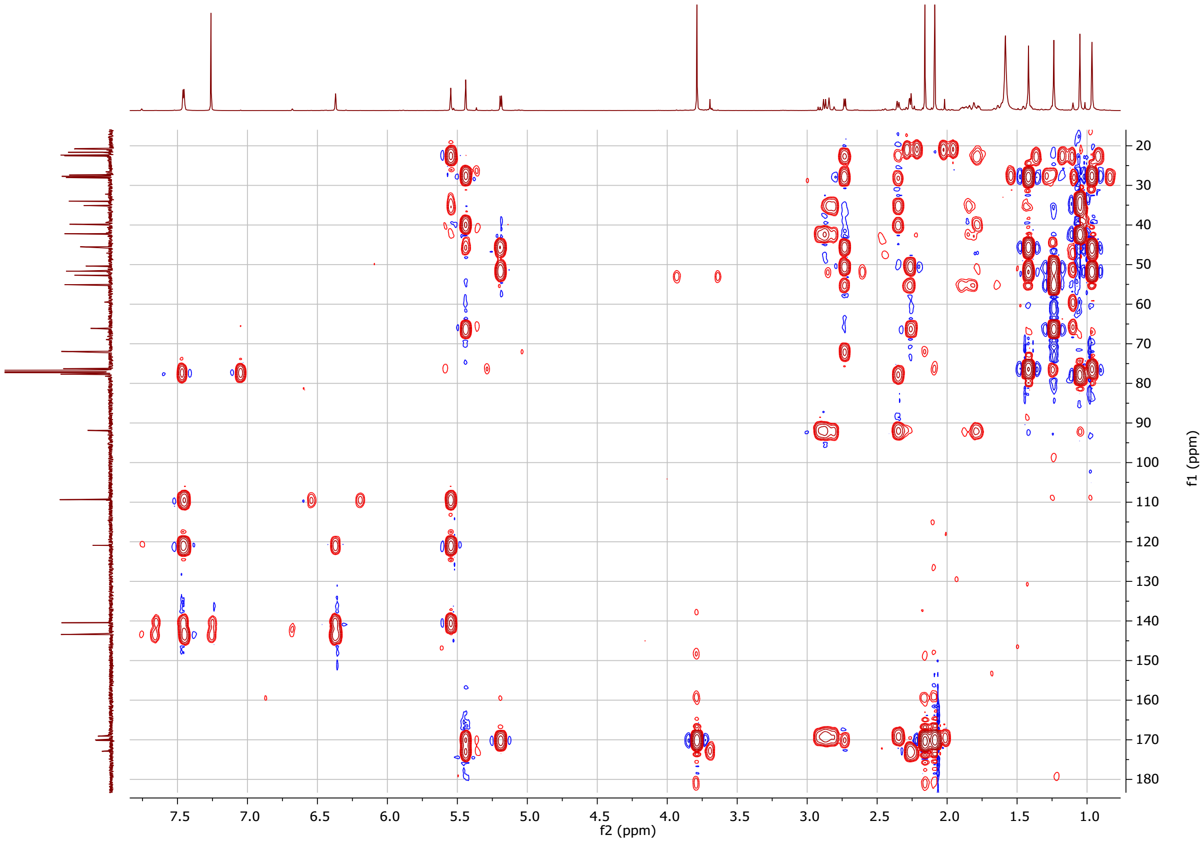

Figure S34. HMBC spectrum of swetemicrolide D (**4**) in $CDCl_3$

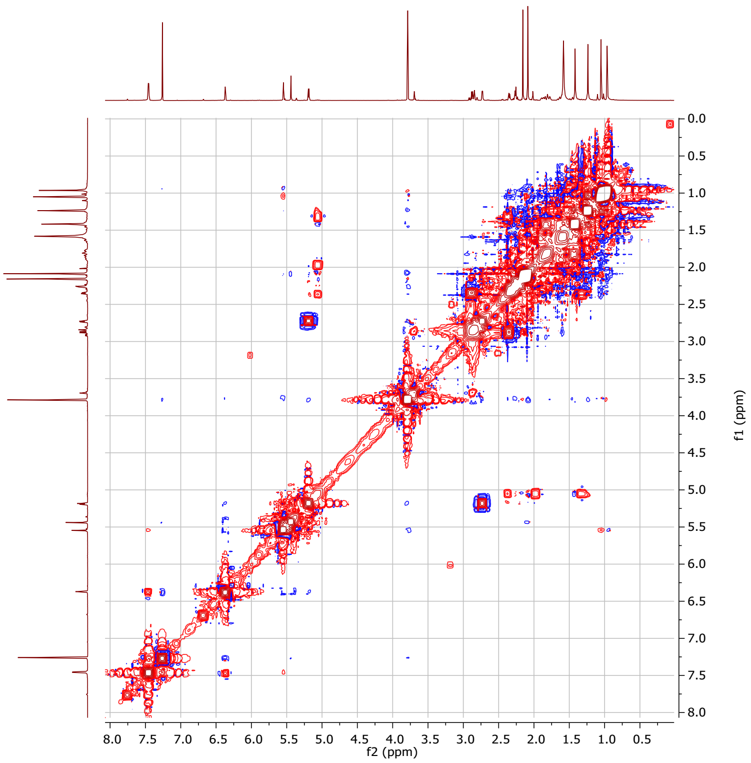

Figure S35. ^{1}H – ^{1}H COSY spectrum of swetemicrolide D (**4**) in $CDCl_3$

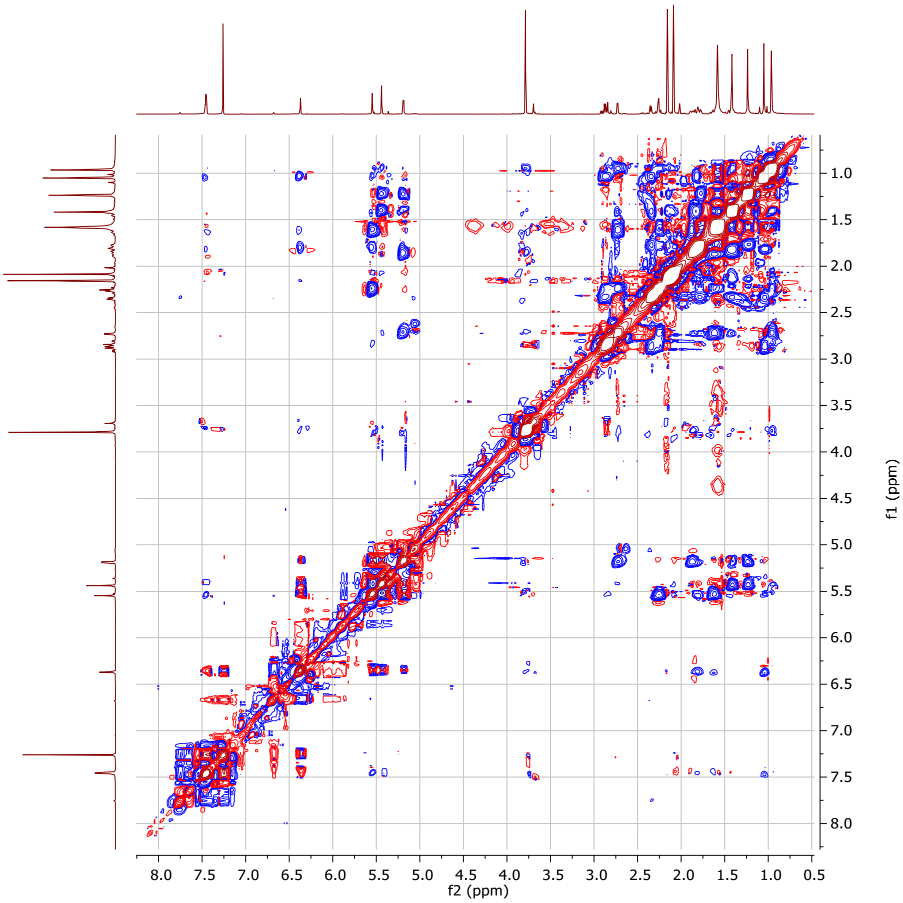

Figure S36. NOESY spectrum of swetemicrolide D (**4**) in $CDCl_3$

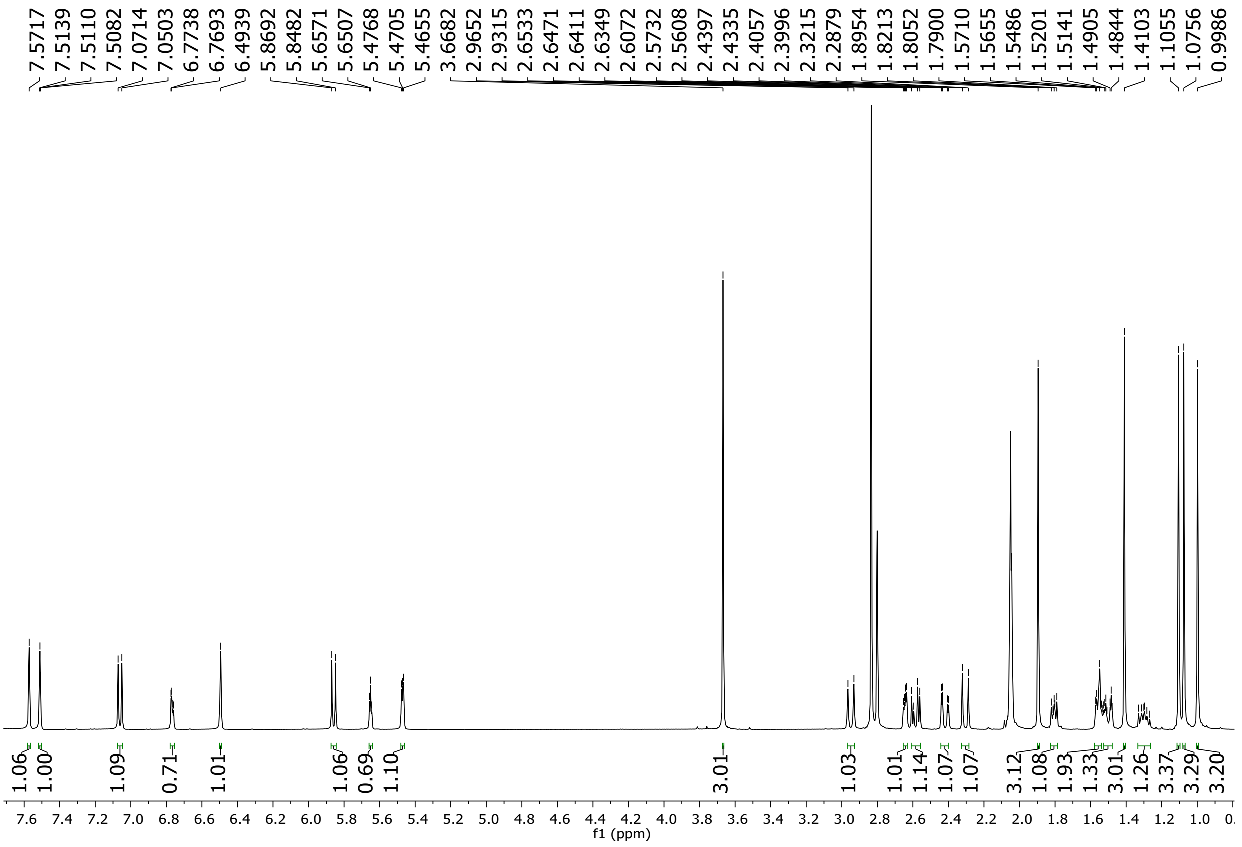

Figure S37. ^{1}H NMR spectrum of swiemahogin A (**5**) in acetone-d_6 (500MHz)

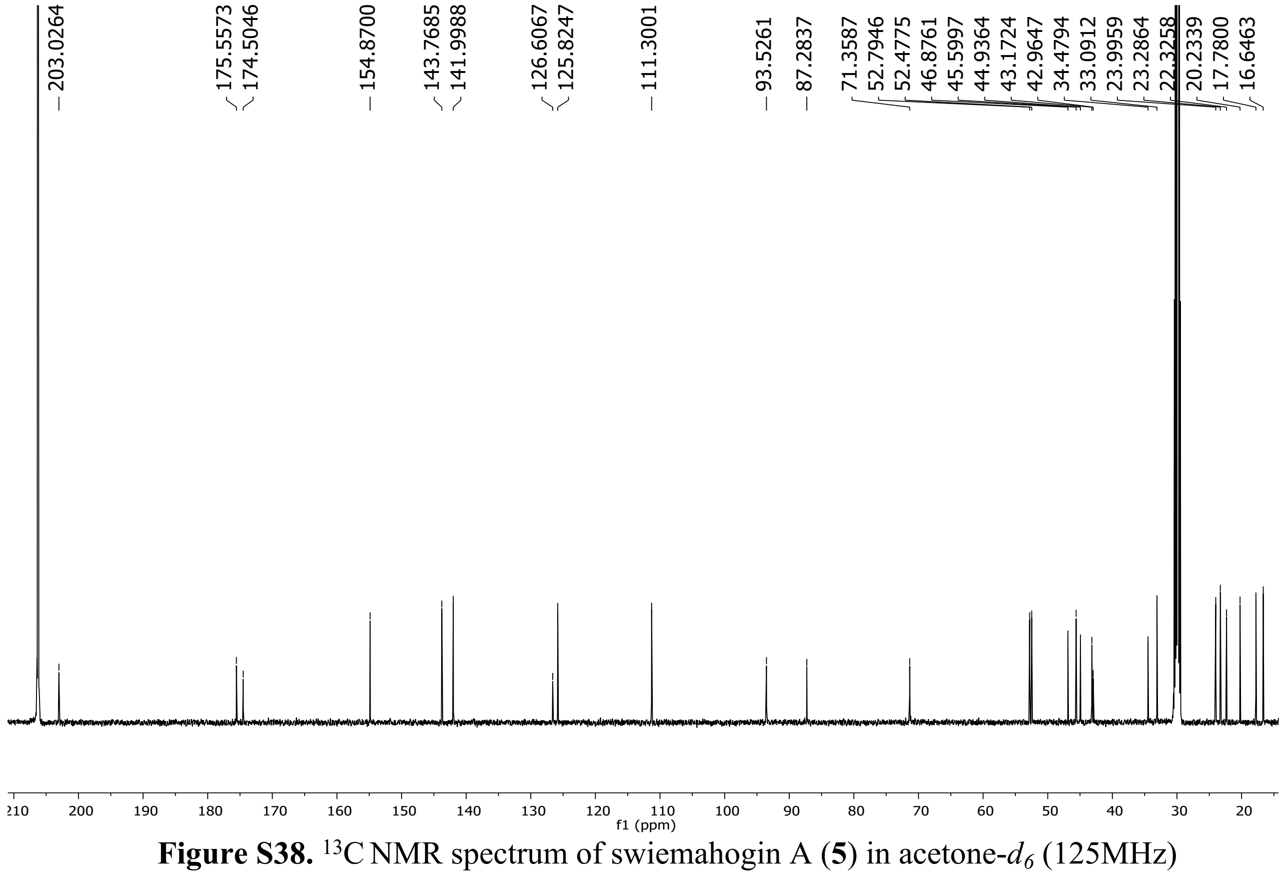

Figure S38. ^{13}C NMR spectrum of swiemahogin A (**5**) in acetone-d_6 (125MHz)

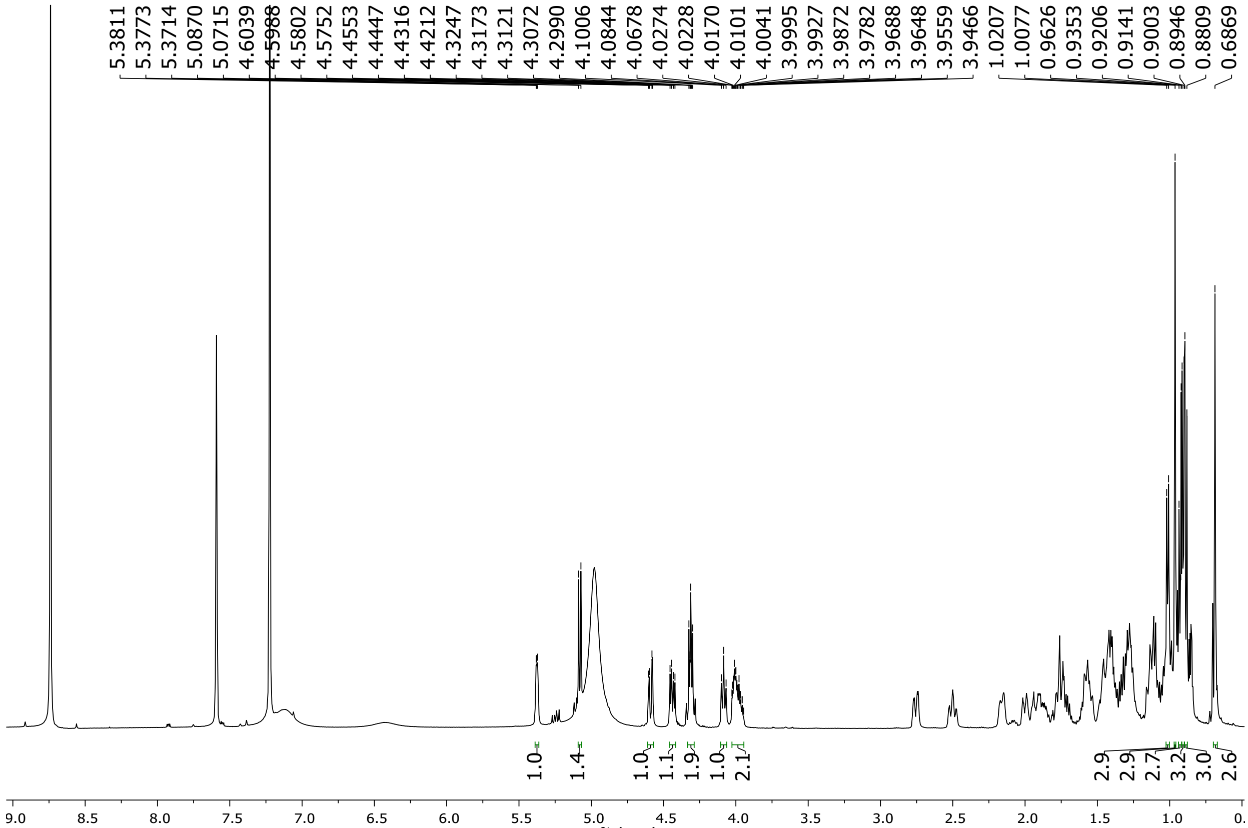

Figure S39. ^{1}H NMR spectrum of *β*-sitosterol-3-*O*-*β*-D-glucopyranoside (**6**) in pyridine-d_5 (500MHz)

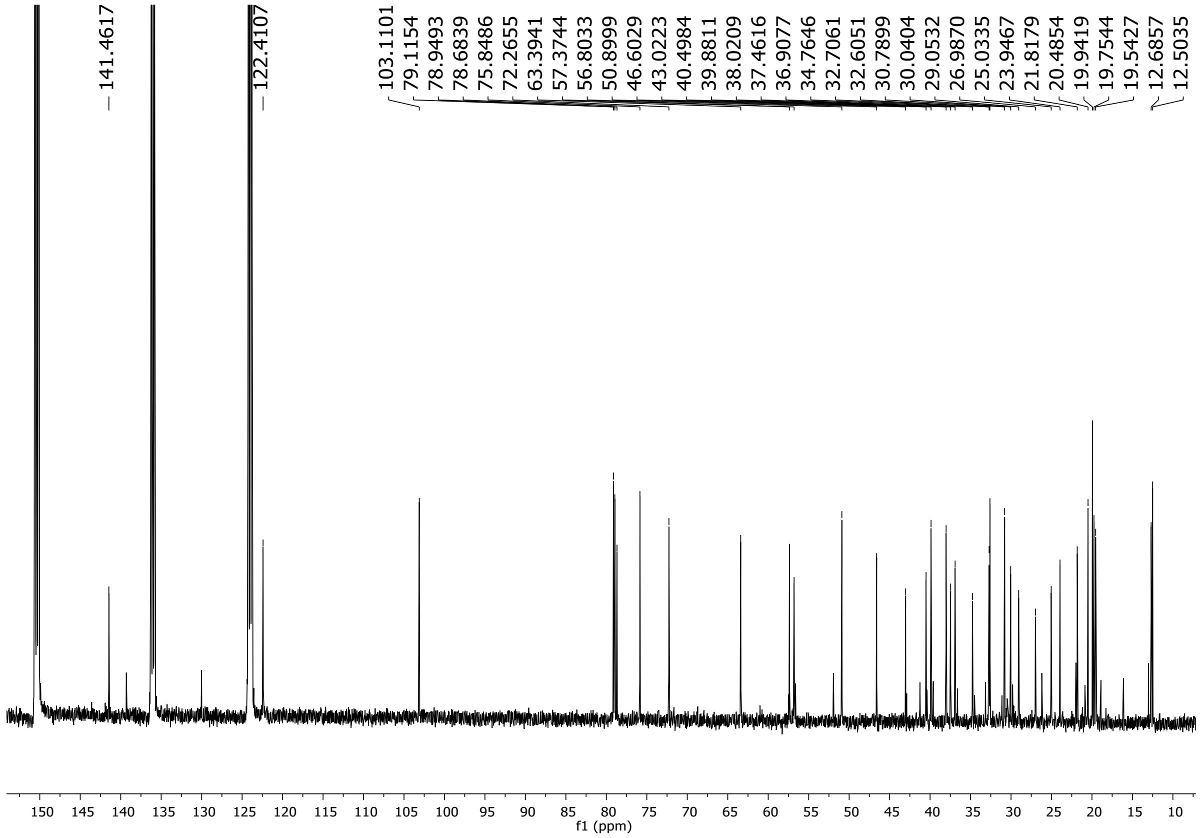

Figure S40. ^{13}C NMR spectrum of *β*-sitosterol-3-*O*-*β*-D-glucopyranoside (**6**) in pyridine-d_5 (125MHz)

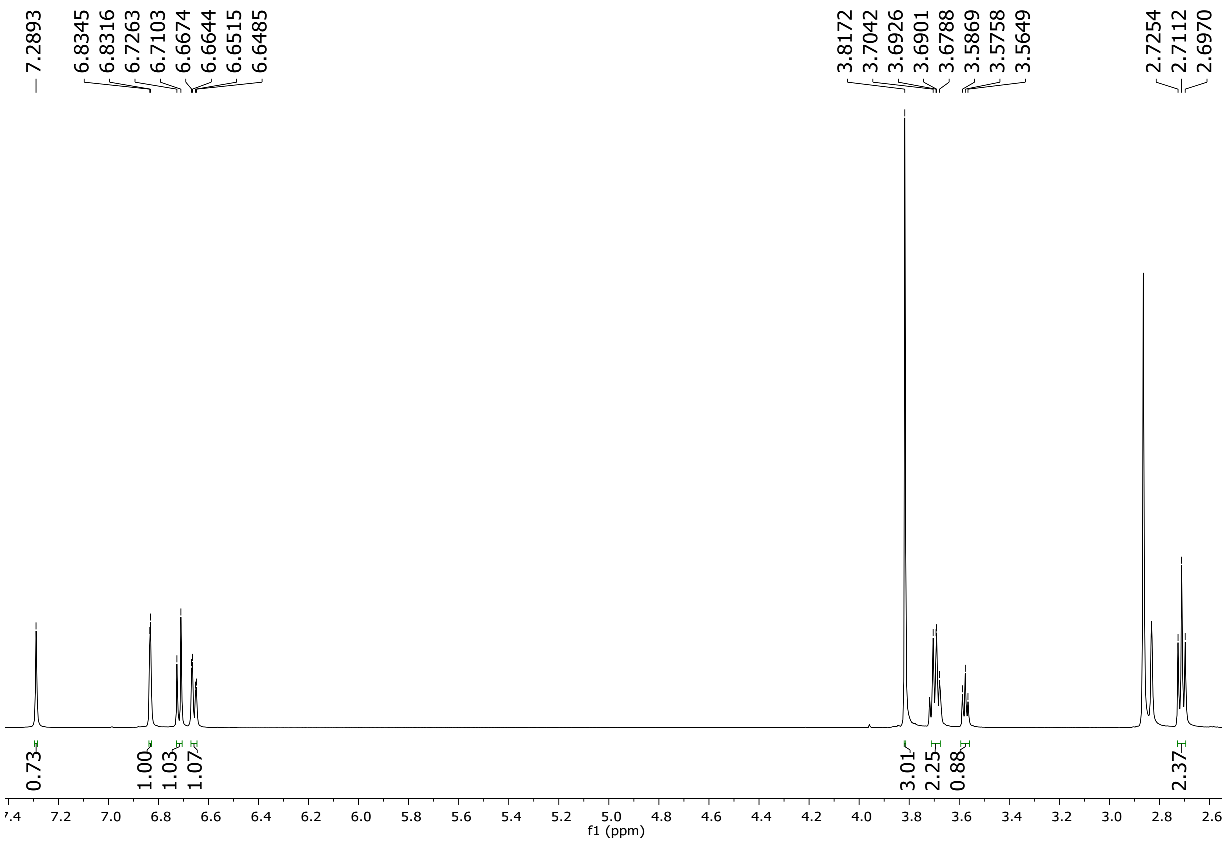

Figure S41. ^{1}H NMR spectrum of homovanillyl alcohol (**7**) in acetone-d_6 (500MHz)

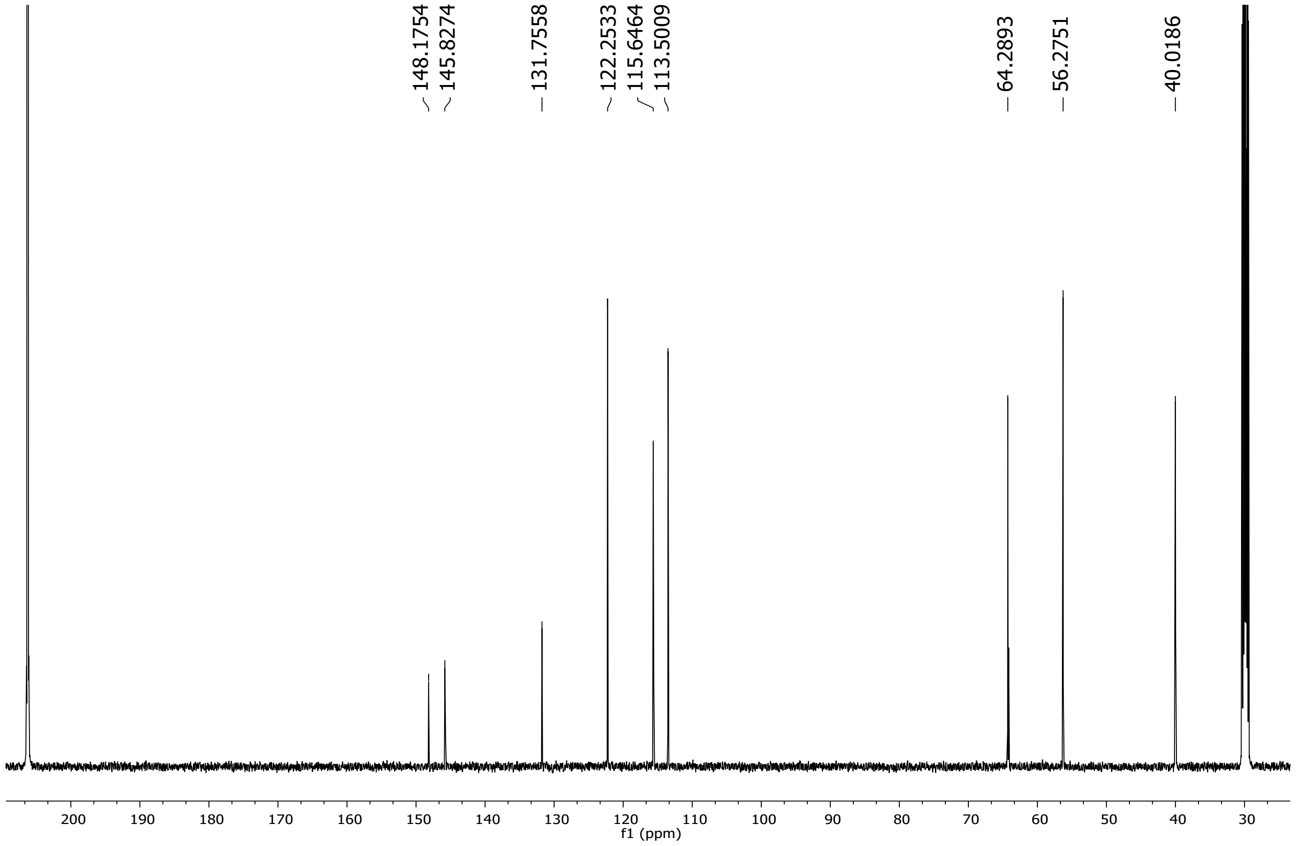

Figure S42. ^{13}C NMR spectrum of homovanillyl alcohol (**7**) in acetone-d_6 (125MHz)
